# Enhanced ion tolerance of electrokinetic locomotion in polyelectrolyte-coated microswimmer

**DOI:** 10.1038/s41467-019-11907-1

**Published:** 2019-09-02

**Authors:** Xiaojun Zhan, Jizhuang Wang, Ze Xiong, Xuan Zhang, Ying Zhou, Jing Zheng, Jianan Chen, Shien-Ping Feng, Jinyao Tang

**Affiliations:** 1 Department of Chemistry, The University of Hong Kong, Hong Kong, 999077 China; 2 Department of Mechanical Engineering, The University of Hong Kong, Hong Kong, 999077 China

**Keywords:** Colloids, Fluid dynamics

## Abstract

Over the last decade, researchers have endeavored to mimic the naturally motile microorganisms and develop artificial nano/microswimmers, which propel themselves in aqueous media. However, most of these nano/microswimmers are propelled by the self-electrophoretic mechanism, which has one critical incompetency: the inability to operate in a high concentration electrolyte solution, such as the most important body fluid, blood. This ionic quenching behavior is well backed by the classical Helmholtz–Smoluchowski theory and seems to be an insurmountable challenge which has shadowed the otherwise promising biomedical applications for artificial nano/microswimmers. Here, we propose that the active nano/microswimmer’s self-electrophoresis is fundamentally different from the passive nanoparticle electrophoresis. By significantly increasing the Dukhin number with polyelectrolyte coating and geometry optimization, a favorable deviation from the Helmholtz–Smoluchowski behavior can be realized, and ion tolerance is enhanced by over 100 times for a visible light-powered self-electrophoretic microswimmer.

## Abstract (web summary)

Electrophoretic microswimmers aren’t well suited to function in vivo because electric-field screening reduces their speed in higher ionic-strength solutions. Here the authors propose a microswimmer that exploits high surface conductance to improve this ion tolerance to enable operation in a high salt environment.

## Introduction

Many microorganisms have evolved with fascinating swimming capabilities which help them to find nutrient or avoid predators. Surprisingly, diverse strategies have been adopted by nature to mediate the locomotion of prokaryotes in different enviorment^1^. The early explanation of those prokaryotes locomotion is proposed by Peter Mitchell who suggested the flagella-assisted self-electrophoresis mechanism^2^. While this mechanism is largely falsified by the remarkable discovery of rotatory flagellar motor^3^, it does inspire the later development of artificial microswimmer counterparts which proved the effectiveness of the self-electrophoresis mechanism in aqueous solution^4,5^. With the recent development of nanotechnology, interest has been grown to mimic the natural motile bacteria and develop artificial nanorobot for the long-envisioned applications such as in healthcare^6–8^, manufacturing^9^, environmental remediation^10,11^, and beyond. However, several major challenges are preventing the development of the fully biocompatible nano/microswimmers, such as low efficiency, high toxicity, and incompatible with electrolyte solution^12^. Most importantly, according to the fundamental electrokinetic theory, the incompatibility with the salty solution is the intrinsic feature of the electrophoresis mechanism, which shadows development toward biomedical application for electrophoresis based microswimmers. Currently, the mainstream microswimmers are developed based on electric field-driven mechanism, which includes self-electrophoresis^13,14^ and electrolyte self-diffusiophoresis^15,16^ as they offer more design flexibility for functional nanorobots, such as programmable chemotaxis or phototaxis^17–21^. However, due to the electric-field screening by the ions, these microswimmers are predicted inherently susceptible to speed reductions in higher ionic-strength solutions^22^, thus not compatible with the in vivo environment. The question may arise here is whether this incompatibility with electrolyte solution is genuinely intrinsic to active swimmer particles, which means all biomedical nanorobots have to be redesigned based on nonelectric field-driven mechanism, such as nonelectrolyte self-diffusiophoresis^23,24^, bubble propulsion^25,26^, self-acoustophoresis^27,28^, self-thermophoresis^29,30^, biohybrid^31,32^, and magnetic field driven^33,34^.

In this paper, we propose a modified electrokinetic model for self-electrophoresis microswimmer which emphasized the importance of the generally ignored surface conductance in microswimmer locomotion and demonstrate a general strategy to improve the ion tolerance of self-electrophoretic locomotion in the electrolyte solution, which is a major advent toward biocompatible nanorobot.

## Results

### Ion tolerance and Dukhin number

First, we propose to quantify the ion tolerance of any nano/microswimmers as “Median Effective Ionic Strength” (*EI*_*50*_) which is defined as the ionic strength of a solution in which the migration speed of the nano/microswimmer is reduced by 50% compared to its migration speed in solution without external electrolytes. Since the *EI*_50_ of all electric field-driven nano/microswimmers are very low (*EI*_50_ < 0.1 mM)^22^, those swimmers cannot be applied to the biological fluid, which significantly limits their potential application. It is critical to investigate whether it is possible to overcome this “ion tolerance challenge”^5,22,35^ and significantly increase the *EI*_50_ of electric field-driven nano/microswimmers for biological application.

Based on the classical Helmholtz–Smoluchowski equation, this poor ion tolerance can be well explained and verified experimentally^13,14^. In fact, the ion quenching has been theoretically concluded as an intrinsic limitation for electrophoresis mechanism^22^ and used as a testimony to prove the electrophoresis mechanism^13,14,36^. However, as initially derived by Smoluchowsiki and re-emphasized later^37–40^, the Helmholtz–Smoluchowski equation ignores the surface conductance and is only valid under low surface conductivity and hard surface condition, while deviation is expected in high surface conductivity and porous surface condition. In practice, the importance of surface conductivity can be quantified by dimensionless quantity Dukhin number (*Du*)^41^ defined as $Du \equiv \frac{{K^\sigma }}{{aK^L}}$, where *K*^*σ*^ is the surface conductivity, *K*^*L*^ is the bulk conductivity of the medium and “*a*” is the characteristic size of the particle. However, since the *Du* is very small ($Du \ll 1$) in almost all experimental condition (*Du* is only significant for nanoparticles smaller than 10 nm), the importance of the surface conductivity is largely overlooked by experimentalists. As demonstrated in this paper, the high-*Du* condition can be realized by grafting the nano/microswimmer with highly ion conductive polyelectrolyte coating which leads to a favorable and surprisingly significant deviation from the classical Helmholtz–Smoluchowski like behavior. By applying the surface polyelectrolyte coating and geometry optimization, over 100 times enhancement of *EI*_50_ in self-electrophoresis based microswimmer can be realized which enables the operation of microswimmers in phosphate-buffered saline (PBS) solution together with live human cells. Since this strategy is addressing the fundamental electrokinetic theory, it can be generally applied to all electric field-driven nano/microswimmers, which enables a whole category of in vivo applications and represents an important advance towards the functional fantastic-voyage like nanorobot. Furthermore, given the diversity of the swimming strategies in natural prokaryotes and this new theory, the Mitchell’s orginal electrophoresis senanior maybe reconsidered and might be veridical for some microorganisms, which may explain the longstanding mystery of how marine *Cyanobacterium synechococcus* swims without flagella^1,42,43^.

### Microswimmer synthesis and electrochemistry characterization

**Fig. 1:**
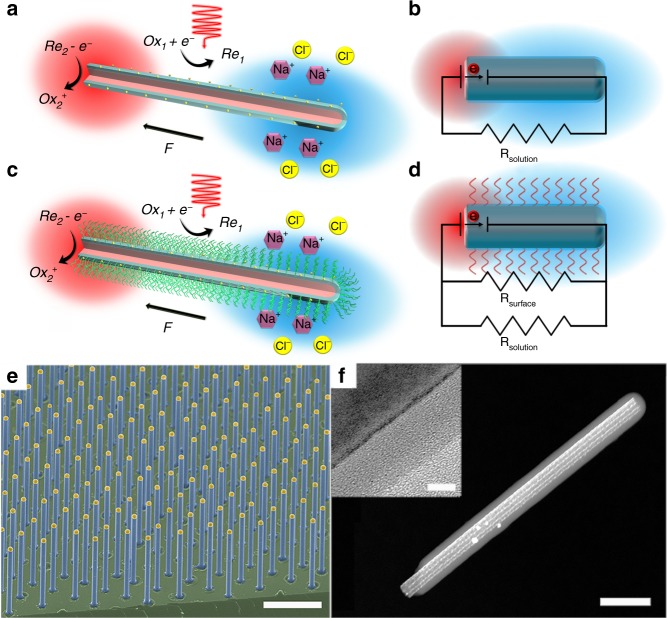
Schematic diagram and structural characterization of microswimmers. **a**, **c** Schematic diagram of silicon nanowire microswimmer without and with polyelectrolyte coating. The SPS layer was chemically grafted onto the core–shell silicon nanowire. Upon illumination, the surface electrochemical reaction driven by the nanowire solar cell produces OH^−^ and H^+^ ions on the n-type silicon shell and exposed p-type silicon core, respectively. The electric field built by the unbalanced ions propel the negatively charged nanowire. **b**, **d** The equivalent circuits of nanowire microswimmer operation without and with polyelectrolyte coating. **e** False-colored scanning electron microscopy image of silicon nanowire array grown by the VLS method. **f** Scanning transmission electron microscopy (STEM) image of SPS-grafted silicon nanowire. Inset is high-resolution TEM image of SPS-modified silicon nanowire. (Scale bars in **e**, **f**, and **f** inset are 10 μm, 200 nm, and 5 nm, respectively)

In this study, our previously reported p/n junction silicon nanowire microswimmer was selected as the model system since its migration can be easily modulated with controlled illumination and its well-established self-electrophoresis mechanism^14^ (Fig. 1a). As shown in Fig. 1c, the silicon nanowire microswimmer was coated with highly ion conductive polyelectrolyte layer, while a highly reversible redox shuttle 1,4-benzoquinone/hydroquinone (BQ/H_2_Q) was utilized to support the redox reaction for self-electrophoresis mechanism. Upon light illumination, the photovoltage generated across the p/n junction drives the electrochemical reaction and produces H^+^ and OH^−^ ions on the exposed p-type silicon core and n-type silicon shell, respectively^14^. The self-generated electric field formed by the unbalanced ion gradient propels the negatively charged nanowire in an electrophoretic manner. With the polyelectrolyte coating (sulfonated polystyrene (SPS) in this case), the highly charged polymer coating serves as the primary ion conductive channel for the nanowire microswimmer. Since the fundamental reason of the self-electrophoresis quenching by external ions relies on the collapsing of the Debye layers in the electrolyte solution, the polyelectrolyte coating on the surface of microswimmer can be regarded as a porous scaffold to support the surface conductive “Debye layers”, which largely prevents the quenching of the electrophoresis. In other words, for uncoated microswimmer, the surface conductivity becomes insignificant compared to the bulk solution conductivity with external electrolyte, which can be presented as low Dukhin number ($Du \ll 1$). With the polyelectrolyte coating, the surface conductivity is supported by the highly conductive polymer, which boosts the *Du* significantly even with external electrolyte. This comparison can also be illustrated as Fig. 1b, d, where the microswimmer operation is modeled in equivalent circuits as a short-circuited battery^44^. Compared to the uncoated microswimmer, the polyelectrolyte-coated microswimmer can be regarded as a solar cell with additional parallel resistor $\left( {R_{\mathrm{surface}}} \right)$. With the surface conductance dominating the ion conductance tangential to the microswimmer, the electric field is less susceptible to the electrolyte concentration in bulk solution.

Our nanowire microswimmer was based on silicon nanowire array prepared by the vapor–liquid–solid method (Supplementary Note 1), which provided uniform nanowire geometry (Fig. 1e). The microswimmer coated with SPS layer was characterized by scanning transmission electron microscopy as shown in Fig. 1f, displaying the uniformly grafted SPS layer onto the nanowire surface.

**Fig. 2:**
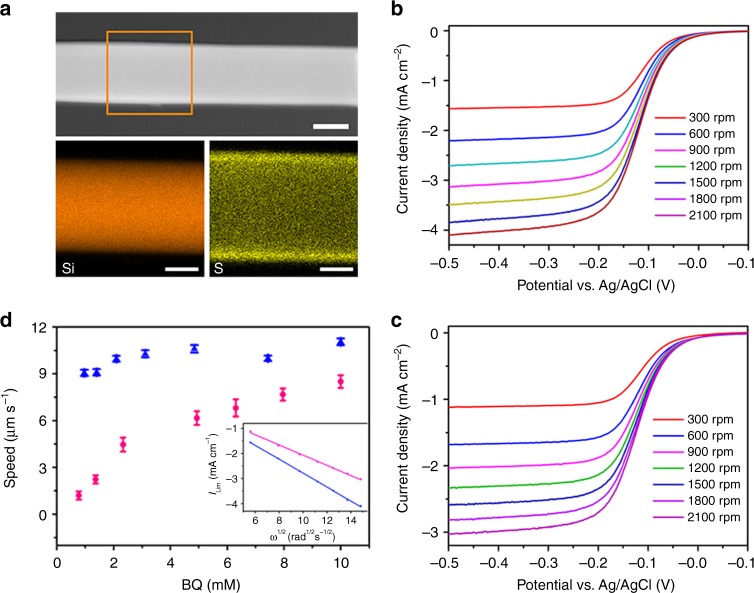
Element analysis and electrochemical characterization of the microswimmers. **a** STEM and Energy-dispersive X-ray spectroscopy (EDX) mapping images of the SPS coated silicon nanowire; scale bar is 50 nm. The brown window in STEM image indicates the elemental mapping area. **b**, **c** Rotating disk electrode (RDE) voltammogram of the bare silicon and the SPS-coated silicon wafer in 2 mM BQ and 1 M KCl aqueous solution. The scanning rate is 10 mV s^−1^. Rotation speeds are 300, 600, 900, 1200, 1500, 1800, and 2100 rpm, respectively. **d** Migration speed vs. the BQ’s concentration of bare microswimmer (blue) and SPS coated microswimmer (red). The inset is the Levich plot of the bare silicon (blue) and the SPS coated silicon wafer (red) at −0.5 V vs. Ag/AgCl

The SPS grafting procedure was developed based on atom-transfer-radical-polymerization (ATRP) and following sulfonation^45^ (Supplementary Note 2). Briefly, the silicon nanowire was activated by immersing in fresh piranha solution for 10 min, which generated –OH terminated silicon surface. After anchoring the 3-bromopropyltrichlorosilane onto the surface by gas phase reaction, the styrene monomers can be polymerized on the silicon surface with the ATRP method. Finally, the grafted polystyrene layer was sulfonated in acetyl sulfate for 5 h at 60 °C and neutralized to sodium polystyrene sulfonate. The relationship between the thickness of grafted layer and polymerization time was determined by monitoring the particle size increases with dynamic light scattering (DLS), which suggested a fast initial polymer growth and saturation at ~100 nm after 10 h reaction (Supplementary Note 3). The energy-dispersive X-ray spectroscopy) mapping images (Fig. 2a) of the SPS-grafted silicon nanowire also confirmed the uniform SPS coating over the entire nanowire surface. Upon SPS coating, the zeta potential of silicon nanowire was changed from −30 ± 13 to −160 ± 32 mV in low-ionic strength condition and neutralized toward higher electrolyte concentration (Supplementary Note 4).

Since the surface redox reaction is essential to the propulsion of the microswimmer, the diffusion of the redox species through the coated SPS layer may substantially limit the electrochemical reaction and affect the migration of microswimmer. To evaluate the influence of the SPS coating on the electrochemistry in our system, we compared the electrochemical kinetics with rotating disk electrode (RDE) voltammetry (Supplementary Note 5) on the bare and the SPS coated silicon electrode as shown in Fig. 2b, c. Levich analysis of the voltammograms, obtained from varieties of rotation speeds (*ω*), yields the diffusion coefficients of BQ on the bare silicon surface ($D_{Si}$) and SPS-coated silicon surface ($D_{sps}$): $D_{Si} = 7.03 \times 10^{ - 6}\,{\mathrm{cm}}^2\, {\mathrm{s}}^{ - 1}\,{\mathrm{and}}\,D_{sps} = 4.43 \times 10^{ - 6}\,{\mathrm{cm}}^2\, {\mathrm{s}}^{ - 1}$ (inset of Fig. 2d). This result indicates that the surface electrochemical reaction is indeed slowed down by the additional SPS coating, but the influence is limited. The effect of this additional diffusion barrier can also be observed in microswimmer’s migration behavior. Figure 2d shows the microswimmer migration speed in solution with different BQ concentrations. Due to faster mass transport, the migration speed of the bare silicon microswimmer is insensitive to the BQ concentration across the measured concentration range, while the speed of the SPS-coated microswimmer depends positively on the BQ concentration and gradually saturated at around 8–10 mM, which implies that higher BQ concentration is needed to reduce the concentration polarization due to slower diffusion through the SPS layer. To mitigate the diffusion influence on microswimmer speed, high BQ concentration (10 mM) was selected in the following experiments.

### Ion tolerance enhancement with polyelectrolyte coating

**Fig. 3:**
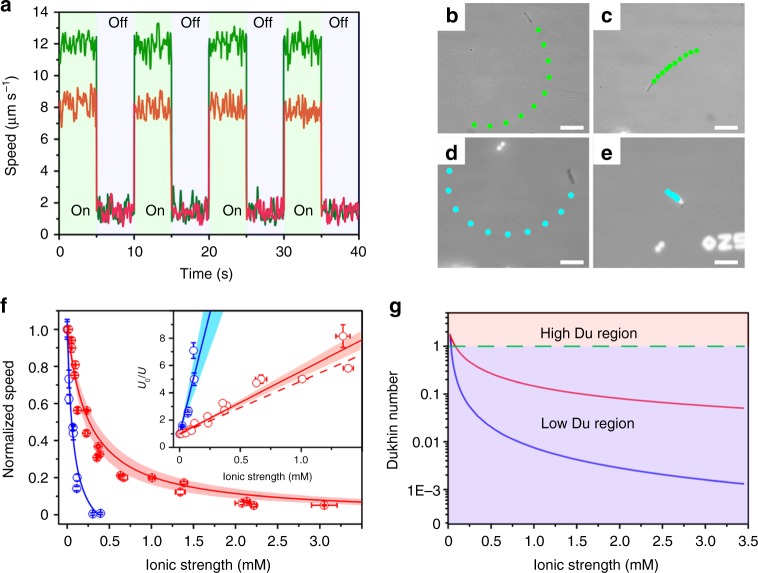
Ion tolerance enhancement for silicon nanowire microswimmer. **a** A typical migration speed of microswimmer with and without SPS coating in BQ/H_2_Q solution under chopped illumination. Microswimmer’s trajectory in BQ/H_2_Q solution: the SPS coated without (**b**) and with (**c**) 0.32 mM NaCl; bare without (**d**) and with (**e**) 0.32 mM NaCl. Scale bar is 20 μm. **f** The dependence of the normalized speed of the bare (blue) and the SPS coated (red) nanowire microswimmer to the solution ionic strength and the corresponding speed is shown in Supplementary Figs. 12 and 13. The solid lines are calculated by our proposed model with measured conductivity, and the band is at 95% confidence interval. The inset shows the linear relationship between the normalized reciprocal speed and the electrolyte concentration as $\frac{{U_0}}{U} = 1 + \frac{1}{{EI_{50}}}C$. The red dash line in the inset is predicted by our model while the solid lines and corresponding color bands indicate the fitting plots and a confidence interval at 95%. **g** The Dukhin number vs. ionic-strength plot for bare microswimmer (blue line) and SPS-coated microswimmer (red line)

Since the nanowire geometry also plays a vital role in ion tolerance which will be discussed in the later section, the geometry of the nanowire under investigation in this section was kept at ~600 nm in diameter and ~15 µm in length. All tests were performed on a customized testing stage equipped with an in-situ electrical conductivity meter, while a halogen lamp equipped with 450 nm long-pass filter was utilized as illumination source with an illumination intensity of ~420 mW cm^−2^ (Supplementary Note 6). Figure 3a shows the migration speed of the bare and the SPS-grafted microswimmer in BQ/H_2_Q solution without the addition of NaCl under chopped illumination (Supplementary Video 1). With the additional SPS coating, the migration is slightly slower than the bare microswimmer, which may be attributed to the slower diffusion of BQ through the SPS layer. Figure 3b, c, d, e shows the trajectories of the microswimmers with and without SPS coating in solution before and after the addition of NaCl (Supplementary Video 2). For the bare microswimmer (Fig. 3d, e), its migration is completely quenched by NaCl with the ionic strength of 0.3 mM. In contrast, the SPS coated microswimmer with comparable speed before the addition of NaCl retained ~35% of its original speed after NaCl addition, which indicates the enhanced ion tolerance.

To quantify the ion tolerance, the same individual microswimmer’s migration speed was measured in solution with different ionic strength adjusted by NaCl (Fig. 3f). The normalized speed of the bare microswimmer (blue) shows a typical reciprocal correlation with the ionic strength of the solution, which is consistent with previously reported result^14,22^, the ion tolerance of bare silicon nanowire microswimmer can be quantified as $EI_{50}^{{\mathrm{Bare}}} = 0.028 \pm 0.009\,{\mathrm{mM}}$ (95% confidence interval). In contrast, for the SPS coated microswimmer, the surface conductivity is significantly enhanced which dramatically promotes the *Du* (Fig. 3g). Since the migration speed relies on solution conductivity as well as microswimmer surface conductivity (*K*^*σ*^), the deviation from the traditional reciprocal relationship and enhanced ion tolerance is observed.

It is worth mentioning that the polyelectrolyte coating induced surface conductivity also significantly attenuates the self-generated electrophoretic field similar to the solution conductivity. However, the charge associated with the polyelectrolyte coating increases the net charge of the microswimmer which largely cancels out the effect of the attenuated electric field. Since the surface conductivity (*K*^*σ*^) of the coated polyelectrolyte is proportional to the surface charge density, the migration speed *U* is proportional to the product of the surface charge and electrophoretic field $U \propto \frac{{K^\sigma }}{{K^\sigma + aK^L}}$ (Supplementary Note 7), where “*a*” is geometry factor defined in *Du* which will be discussed in later section. This equation can be further simplified into Eq. (1) by normalizing with initial migration speed *U*_0_, which is a general equation applied to all *Du* condition.

1 $$\frac{U}{{U_0}} = \frac{{K^\sigma }}{{K^\sigma + aK^L}} = \frac{{Du}}{{1 + Du}}$$

In Eq. (1), when the *Du* is small ($K^\sigma \ll aK^L$), the migration speed shows reciprocal dependence on $K^L$ as $\frac{U}{{U_0}} = \frac{{K^\sigma }}{{K^\sigma + aK^L}} \approx \frac{{K^\sigma }}{{aK^L}}\left( {Du \ll 1} \right)$, which aligns with classical Helmholtz–Smoluchowski equation; while when *Du* is large ($K^\sigma \gg aK^L$), the migration speed is independent of the solution conductivity and not quenched by the high surface conductivity as $\frac{U}{{U_0}} = \frac{{K^\sigma }}{{K^\sigma + aK^L}} = \frac{{Du}}{{1 + Du}} \approx 1(Du \gg 1)$. Apparently, with the much-enhanced surface conductivity, the migration speed is no longer inversely proportional to the solution conductivity (*K*^*L*^). Generally, for any microswimmer, a more elegant way to show the ion tolerance enhancement is plotting the normalized reciprocal speed (*U*_*0*_/*U*) as a function of electrolyte concentration (*C*). As shown in Eq. (2), a linear relationship is expected with its slope equals to the reciprocal of its ion tolerance (1/*EI*_50_), where $\Lambda _m$ is the molar conductivity of the added electrolyte.

2 $$\frac{{U_0}}{U} = 1 + \frac{1}{{EI_{50}}}C{.}$$

3 $$EI_{50} = \frac{{K^\sigma }}{{a\Lambda _m}}.$$

As shown in the inset of Fig. 3f, the experimental results confirmed this linear relationship while the enhanced *EI*_50_ is clearly observed with a smaller slope. Although this simple equation is merely a rough estimation and many additional corrections are needed to improve accuracy like in early studies^38–40^, it does illustrate the important electrokinetic physics of the nano/microswimmer under high-*Du* condition. It is worth noting that this ion tolerance enhancement is intrinsically related to the surface conductivity of microswimmer instead of the substrate surface conductivity as ion tolerance enhancement of bare microswimmer on SPS-coated glass substrate was not observed (Supplementary Note 8).

To evaluate this model quantitatively, the key value needed is the surface conductivity *K*^*σ*^ which determines the *Du* and the ion tolerance. We placed a glass slide inside the reaction flask together with the nanowire wafer and measured the surface conductivity of the coated SPS layer with impedance measurement (Supplementary Note 9). With this $K^\sigma = (2.18\,0.45) \times 10^{ - 8}$ S·□ (95% confidence interval), the predicted ionic strength measured dependence curve can be plotted using Eqs. (1)–(3). As shown in Fig. 3f (solid red line and red dash line of the inset), the measured migration speed in different ionic-strength solution matches very well with Eqs. (1) and (2). The speed to ionic strength dependence curve obviously shifts to the higher ionic-strength region and reaches about 1.5 mM with ~20% remaining speed for the SPS-coated microswimmer, indicating that the ion tolerance has been improved by ~8 times ($EI_{50}^{{\mathrm{SPS}}} = 0.218 \pm 0.023\,{\mathrm{mM}}$, 95% confidence interval) compared to the bare microswimmer ($EI_{50}^{{\mathrm{Bare}}} = 0.028 \pm 0.009\,{\mathrm{mM}}$, 95% confidence interval). This ion tolerance improvement can also be observed in the normalized reciprocal speed plot (Fig. 3f inset) as a smaller slope. Here, the *EI*_50_ are reported based on typical microswimmer data, while the averaged *EI*_50_ of microswimmer ensemble also show similar ion tolerance enhancement upon SPS coating ($EI_{50}^{{\mathrm{bare}},{\mathrm{ensemble}}} = 0.037 \pm 0.008\,{\mathrm{mM}}$ and $EI_{50}^{{\mathrm{SPS}},{\mathrm{ensemble}}} = 0.232 \pm 0.064\,{\mathrm{mM}}$, Supplementary Note 10). As a side note, in our model, the *EI*_50_ is proportional to the surface conductivity, as the SPS coating enhanced the surface conductivity by over 1000 times, in principal, the *EI*_50_ should also enhanced proportionally. However, since in the self-electrophoresis microswimmer scenario, the electric field is localized around the microswimmer reaction site instead of all over the particle as in traditional electrophoresis experiment, an effective size instead of geometric size should be used to calculate the *Du*, which results in a much smaller effective size for bare microswimmer and greatly cancels the surface conductivity enhancement in SPS-coated microswimmer (Supplementary Note 11). This geometric size effect fundamentally distinct the active microswimmer particle from the traditional passive particle, and will be further elaborated in next section. However, the importance of the ion tolerance enhancement by surface conductivity should not be undermined.

Fundamentally, all electrokinetic phenomenon is originated from the ionic flow through the interfacial double layer. In this sense, the efficiency of the electrokinetic phenomenon including the electrophoresis and electrolyte diffusiophoresis is primarily determined by the *Du*, which quantifies the relative importance of the surface over bulk conductivity. Figure 3g shows the *Du* of coated and uncoated microswimmer with same geometry (length: 15 μm, diameter: 600 nm). In electrolyte solution, the bare microswimmer has *Du* ≪ 1, which means the majority of the ions flow through the bulk solution far away from the microswimmer and does not provide electro-osmosis flow (EOF), thus no propulsion force is generated. With the porous ion conductive coating, the *Du* is much enhanced, and the ionic flow is primarily through the conductive coating and can generate EOF and contributes to the microswimmer propulsion.

**Fig. 4:**
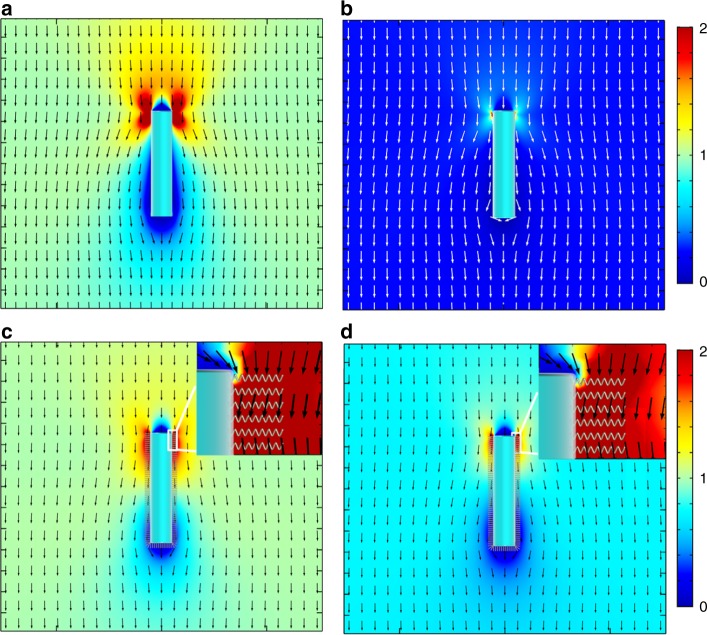
Numerical simulation generated plots of the EOF velocity field around the microswimmers. The anode located on the top of the nanowire and all the other surfaces are cathodes as described in Fig. 1a. **a**, **b** The EOF velocity field around the bare nanowire without and with NaCl (0.166 mM), respectively. **c**, **d** The EOF velocity field around the SPS modified nanowire without and with NaCl (0.166 mM), respectively. The insets show the detailed EOF field inside the SPS-coating layers. Colors in all images represent the EOF speed normalized by the flow speed far away from the nanowire without NaCl, and arrows denote flow direction. To facilitate the comparison, the color scale is the same for the same nanowire

To further illustrate the ion tolerance enhancement of SPS coating on the microswimmer, Fig. 4 shows the numerical simulation results of EOF velocity field around the coated and uncoated microswimmer in solution with different ionic strength (Supplementary Note 12). In this model, the SPS coating is treated as a porous media with finite ion conductivity where the EOF can be generated inside^46^. This EOF penetration assumption means the porosity of the grafted SPS layer should be sufficiently low^39^, while the dense polyelectrolyte coating should be avoided. Before adding the NaCl, the tangential EOF is similar for both microswimmers regardless of its coating status (Fig. 4a, c). As expected, by adding 0.166 mM NaCl, the EOF around the bare silicon is almost completely suppressed due to the ionic screening of the electrophoretic field (Fig. 4b). In contrast, although the electrophoretic field is also suppressed in the microswimmer with SPS coating, a nontrivial EOF is observed inside the SPS layer (Fig. 4d), which supports the sufficient propulsion of microswimmer and is the origin of the ion tolerance enhancement. This EOF penetration inside the porous surface layer has been envisioned theoretically for decades^39,40,46^, and was utilized to explain the deviation from the classical Helmholtz–Smoluchowski model in the electrokinetic experiment. However, since the predicted and observed deviations are relatively small, these corrections are not widely accepted and tested experimentally. On the other hand, for active particle system such as artificial microswimmer, this additional EOF inside the porous layer coating can dominate the propulsion of microswimmer and dramatically enhance the ion tolerance as shown in our experiments. As predicted theoretically^39,40^, the ion tolerance would also depend on the electrolyte type and the porous layer coating type as the interaction potential of different ions with different porous layers vary, which is also observed experimentally (Supplementary Note 13). However, since the primary purpose of enhancing the ion tolerance for the nano/microswimmers is the biological application, where the dominant electrolyte is NaCl and KCl, the microswimmer’s ion tolerance in different electrolytes are not extensively elaborated here. On the other hand, another unique feature for active microswimmer system which has not been envisioned is that the self-generated propulsion electric field is determined by the geometric assembly of anode and cathode, which will strongly modulate the ion tolerance of microswimmers. This geometric dependence will be elaborated in the next section.

### Enhanced ion tolerance with geometry optimization

Although the polyelectrolyte coating can significantly improve the ion tolerance due to the increased surface conductivity, the *EI*_50_ is still too low to be applied to high concentration electrolyte solution as similar to the in vivo environment. To further enhance the ion tolerance, the surface conductance needs to dominate the tangential ion conductance which can be achieved in two ways: (1) further enhance the surface conductivity and (2) adjust the geometry of the microswimmer to reduce the contribution from solution conductivity. Since the SPS grafting chemistry only produces the polymer with length ~100 nm and the possibly even slower diffusion kinetics in the thicker polymer coating layer, we tried to further enhance the ion tolerance of our microswimmer by adjusting the geometry of nanowire (Supplementary Note 14).

**Fig. 5:**
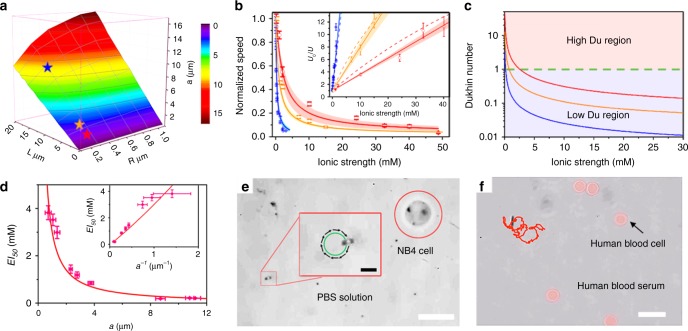
Geometry optimization biocompatibility demonstration with the live human cell (acute promyelocytic leukemia NB4 and human blood cells). **a** The dependence of the geometry factor “*a*” on nanowire’s geometry. The three nanowire geometries in **b** are marked as the red star (short nanowire), orange star (medium nanowire), and blue star (long nanowire) respectively. **b** The representative ionic strength dependence of the normalized speed of the SPS coated nanowire microswimmer for short (red), medium (orange), and long (blue) nanowire microswimmers. The corresponding absolute velocity data is shown in Supplementary Fig. 13. The solid lines are calculated by Eq. (2) with measured SPS conductivity and the bands are the confidence interval at 95% confidence. The inset shows the linear relationship of the normalized reciprocal speed with the ionic strength. The dash lines are the expected relationships predicted by Eq. (2). The solid lines and corresponding color bands in the inset indicate the fitting plots with the confidence interval of the experimental data at 95% confidence level. **c** The Dukhin number to the ionic strength dependence for the SPS-coated microswimmers with different geometries. The red, orange, and blue curves are corresponding to the *Du* of the short, medium, and long nanowire with *a* = 0.74, 2.32, and 9.16 μm, respectively. **d** The relationship between *EI*_*50*_ and geometric factor “*a*”. The dots correspond to data from Table S5. The red line shows the theoretical curve based on Eq. (3). The inset shows the corresponding linear relationship between *EI*_*50*_ and *a*^*−*^^1^. The error bar of “*a*” and *a*^−1^reflects the size uncertainty obtained from the swimmers’ tracking videos; see details in Supplementary Note 15. **e** Demonstration of microswimmer operation in PBS buffer solution together with the live NB4 cell (scale bar is 20 μm). The inset shows the magnified view of the microswimmer with the trajectory of 2 s (scale bar of the inset is 5 μm). **f** The trajectory of the microswimmer operation in diluted human blood (scale bar is 10 μm)

In Eq. (3), the $EI_{50} = \frac{{K^\sigma }}{{a\Lambda _m}}$, which can be increased with higher surface conductivity (*K*^*σ*^) or smaller particle characteristic length (*a*). Although has not been understood, this size-dependent ion tolerance has been observed in the single-enzyme molecule^17^, stomatocyte nanomotors^36^, synthetic vesicles^47^, and mesoporous nanomotor^48^, which suggests that the nanomotor with small characteristic size can tolerate higher ionic strength better than bigger size micromotors. According to the Eq. (3), appreciable ion tolerance enhancement for uncoated microswimmer can only be pronounced with size approaching to tens of nanometers or less which is consistent with previous observation^17,36,47^. Upon surface conductivity dramatically enhanced by polymer coating, this geometric dependence is greatly shifted to larger size and can be observed experimentally. In our nanowire geometry, the geometric related factor “*a*” is calculated by numerical simulation (Supplementary Note 15) and presented in Fig. 5a, which suggests that geometric factor “*a*” is primarily determined by the nanowire length and is only weakly related to the diameter. The SPS-coated nanowires with different diameter and length were measured and the *EI*_50_ were tested corresponding to different geometric factor “*a*”. As shown in Fig. 5b, the ion tolerance of nanowire microswimmers was further enhanced with decreasing nanowire length. For this geometry dependence study, the geometric factor “*a*” is reduced from 9.16 μm (15 μm in length and 600 nm in diameter swimmer) to 2.32 μm (4.3 μm in length and 400 nm in diameter swimmer) and to 0.74 μm (2 μm in length and 400 nm in diameter swimmer), which provides additional *Du* enhancement (Fig. 5c) and additional ~12 times enhancement in ion tolerance. The ion tolerance for the shortest microswimmer is enhanced to $EI_{50}^{{\mathrm{SPS}}3} = 3.820 \pm 0.294\,{\mathrm{mM}}$, which is enhanced by ~17 times over the long nanowire microswimmer. As shown in Fig. 5d, the *EI*_50_ of SPS-coated microswimmers is reversely proportional to the geometric factor “*a*” as predicted in Eq. 3 (Supplementary Fig. 13 and Supplementary Table 5).

As stated previously, contrary to traditional electrophoresis experiment, in self-propelled microswimmer, the electrophoretic field is localized around the reaction electrode^14^ and only part of the nanowire microswimmer is effectively exposed to the electric field. As a result, when calculating the *Du* for self-electrophoresis nanowire microswimmer, only the part exposed to the self-generated electric field should be counted for effective geometric size which could be much smaller than the physical size of the swimmer. This discrepancy is the fundamental difference between the self-electrophoretic active particles to the passive particles in the electrokinetic experiment with applied external field. Even for the same particle, the *Du* in self-electrophoresis propulsion could be dramatically different from the *Du* in electrophoresis experiment. In bare nanowire microswimmer, due to the low-surface conductivity, the electrochemical reaction is much localized to the exposed p/n junction area at low-ionic strength condition and expands with the solution conductivity increase, which is similar to the nanogap electrochemical cell^49^ where the electrochemical reaction is localized within 100 nm distance between the cathode and the anode in pure water. As a result, the fitted geometric size “*a*” of bare nanowire microswimmer is much smaller than the physical size of the nanowire and increases with the solution conductivity (Supplementary Note 11). This electrochemistry localization unified the ion tolerance of bare nanowire microswimmers regardless of their different physical size. Upon the SPS coating, the surface conductivity is dramatically enhanced, which largely cancels this electrochemistry localization and the geometric size effect can be observed. In this argument, the ion tolerance of the electrokinetic locomotion is determined by the *Du* of a microswimmer. However, contrary to the traditional *Du* for the passive particle, the *Du* for active particles is also highly depended on the localized electrophoretic field distribution around the particle, which can dramatically differ from the *Du* measured from electrophoresis experiment. In this regard, the early electrophoresis experiment^43^ is not sufficient to rule out the possible electrophoresis propulsion mechanism of the nonflagellated cyanobacteria^42^, which does require the abundant surface polyelectrolyte coating to swim^50^.

To test the general efficacy of ion tolerance enhancement by surface conductivity coating, we load SPS on two additional widely studied SiO_2_/Pt and TiO_2_/Pt Janus nanomotors which work on electrolyte self-diffusiophoresis^15,16^. Although further optimization is needed, a similar ion-tolerance improvement is also observed for these systems (Supplementary Note 16).

Biocompatibility is a grand challenge for microswimmers and has to be addressed before a genuine in vivo application can be realized. Particularly, biocompatibility contains two aspects^12^: (1) the low toxicity of microswimmer and its supporting chemicals and (2) the high tolerance of the microswimmer to the biological environment. In this paper, we only focus on the ion tolerance in second aspect, while the cytotoxicity of the supporting chemical BQ/H_2_Q is also tested (Supplementary Note 17), which suggested an improved toxicity over H_2_O_2_ and can support the live cells upto a few hours, while still not satisfactory for in vivo application. The nontoxic biocompatible supporting chemicals should be further explored. Nevertheless, it is expected that the electrokinetic model proposed here is general to other electrochemical nano/microswimmers thus it is applicable to enhance the ion tolerance to all electrochemistry driven microswimmers and not related to supporting chemicals. To demonstrate the enhanced ion tolerance, the polyelectrolyte modified microswimmers were transferred into 40 mM PBS buffer solution with BQ/H_2_Q, together with the live model human cell (acute promyelocytic leukemia NB4 cell). As shown in Fig. 5e, a directional migration with a speed of ~4 μm s^−1^ was observed adjacent to a live cell (Supplementary Video 3). The migration of multiple high-ion tolerance microswimmers was also recorded to verify the repeatability (Supplementary Video 4). Another biocompatibility challenge of nano/microswimmers is the biofouling due to the nonspecific protein adsorption. The polyelectrolyte and other nanostructured polymer thin film were widely explored as an antifouling layer in medical devices^51^. We tested the SPS coated microswimmer in human blood medium to demonstrate this antifouling effect. Although further improvement is needed, the SPS coating alleviates the nonspecific adhesion of albumin to the microswimmer surface (Supplementary Note 18), and the SPS coated microswimmers can migrate in the diluted human blood (Fig. 5f) (Supplementary Note 19, Supplementary Video 5). Furthermore, to show the generality of the porous layer coating enhancement, additional porous media coating: Zeolitic imidazolate frameworks (ZIF-8) was utilized as another model to proof the concept of ion tolerance enhancement where directional migration of ZIF-8-coated microswimmer in ~190 mM PBS and diluted human blood were successfully observed (Supplementary Note 20, Supplementary Video 6).

## Discussion

In summary, the fallacious belief that the self-electrophoresis nano/microswimmer cannot operate in high-salt aqueous environments has been disproved theoretically and experimentally. The efficiency of the self-electrophoresis is determined by the Dukhin number which can be enhanced with polyelectrolyte coating. A simple yet powerful quantitative model is proposed to describe the microswimmer ion tolerance enhancement which suggested that both surface conductivity enhancement and geometry optimization can be applied synergetically. Based on this strategy, the ion tolerance of self-electrophoresis microswimmer is dramatically enhanced by over 100 times. We believe this ion tolerance enhancement strategy can also be applied to other existing and envisioned electric field propelled nanomotor system which could be more versatile than nonelectric field-driven nanomotor. After conquering this “ion tolerance challenge”, more novel biological and in vivo nano/microswimmer and nanorobotic systems can be expected.

## Supplementary information

Supplementary Information

Supplementary Video 1

Supplementary Video 2

Supplementary Video 3

Supplementary Video 4

Supplementary Video 5

Supplementary Video 6

## Data Availability

The data that support the findings of this study are available from the corresponding author upon reasonable request.
