# Supplementary material for: Enhanced ion tolerance of electrokinetic locomotion in polyelectrolyte-coated microswimmer
Source: Nat Commun. 2019 Sep 2;10:3921. doi: 10.1038/s41467-019-11907-1 (PMC6718642; doi:10.1038/s41467-019-11907-1)
Supplement: Supplementary file 1 — Supplementary Information [file 41467_2019_11907_MOESM1_ESM.pdf]

Supplementary Information

Enhanced Ion Tolerance of Electrokinetic Locomotion in Polyelectrolyte Coated Microswimmer

Xiaojun Zhan,[1†] Jizhuang Wang,[1†] Ze Xiong,[1] Xuan Zhang,[1] Ying Zhou,[1], Jing Zheng,[1] Jianan Chen,[2] Shien-Ping Feng,[2] Jinyao Tang[1]*

1. Department of Chemistry, The University of Hong Kong, Hong Kong 999077, China.
2. Department of Mechanical Engineering, The University of Hong Kong, Hong Kong 999077, China.

* Correspondence and requests for materials should be addressed to J.T. (Email: jinyao@hku.hk).

† These authors contributed equally to this work.

Table of Contents

Supplementary Figures

Supplementary Figure 1. Sulfonated poly(styrene) (SPS) grafting process.

Supplementary Figure 2. The thickness of polymer coating vs. reaction time measured by DLS in aqueous solution.

Supplementary Figure 3. The zeta potential of silicon nanowires without and with SPS coating in different salt concentrations.

Supplementary Figure 4. The contact angle change of glass substrate in each step of SPS coating.

Supplementary Figure 5. The dependence of the normalized speed of the bare nanowire microswimmer on bare glass and SPS coated glass to the solution ionic strength.

Supplementary Figure 6. SPS surface conductivity measurement using impedance measurement.

Supplementary Figure 7. The dependence of the normalized speed of the bare and the long SPS coated microswimmer (~15 μm) to the solution ionic strength.

Supplementary Figure 8. Schematic diagram of the active area of microswimmer with and without SPS coating.

Supplementary Figure 9. Geometric factor fitting for bare microswimmer and SPS coated microswimmer.

Supplementary Figure 10. The effect of SPS coating on the properties of 15 μm long nanowire microswimmer.

Supplementary Figure 11. The electroosmosis speed vs ionic strength of different electrolytes.

Supplementary Figure 12. The migration behavior of bare microswimmers in different ionic strength solution.

Supplementary Figure 13. The migration behavior of the SPS coated microswimmers in different ionic strength solution.

Supplementary Figure 14. SEM images of TiO_2/Pt and SiO_2/Pt Janus microspheres.

Supplementary Figure 15. Ion tolerance improvement of diffusiophoresis microswimmers.

Supplementary Figure 16. The images of the NB4 cells cultured for 5.5 h with pure culture medium, H_2Q/BQ and H_2O_2.

Supplementary Figure 17. The cell density comparison of NB4 cell treated with H_2Q/BQ and control sample without H_2Q/BQ treatment before and after re-culture 24 h.

Supplementary Figure 18. LD_{50} measurement of U2OS cells over the concentration of H_2Q/BQ.

Supplementary Figure 19. Electrochemistry characterization of the silicon electrodes with and without SPS coating in human blood.

Supplementary Figure 20. The migration trajectory of the same SPS coated microswimmer before and after addition of human blood serum.

Supplementary Figure 21. TEM image and trajectory of ZIF-8 coated silicon nanowire microswimmer.

Supplementary Tables

Supplementary Table 1. List of fitted geometric factor for the bare microswimmers with different geometric size.

Supplementary Table 2. Typical parameters used in the simulations.

Supplementary Table 3. List of parameters used in the proposed equations.

Supplementary Table 4. Geometric parameters and EI_{50} of bare microswimmers.

Supplementary Table 5. Geometric parameters and EI_{50} of SPS coated microswimmers.

Supplementary Notes

Supplementary Note 1. Microswimmer fabrication procedure

Supplementary Note 2. Polystyrene grafting and sulfonating procedure

Supplementary Note 3. SPS thickness characterization

Supplementary Note 4. Zeta potential measurement

Supplementary Note 5. Electrochemistry characterization: RDE voltammetry

Supplementary Note 6. Microswimmer migration measurement procedure and data analysis

Supplementary Note 7. Formula deduction

Supplementary Note 8. Migration behavior of bare microswimmers on the SPS coated substrate

Supplementary Note 9. Impedance measurement of SPS layer

Supplementary Note 10. Ion tolerance improvement of SPS coated microswimmer ensemble

Supplementary Note 11. Geometric factor of active bare and SPS coated microswimmer

Supplementary Note 12. EOF velocity field simulation

Supplementary Note 13. Ion tolerance of EOF for different ions

Supplementary Note 14. Enhanced ion tolerance with geometry optimization

Supplementary Note 15. Geometric factor "a" of SPS coated swimmer

Supplementary Note 16. The ion tolerance enhancement of diffusiophoresis microswimmers

Supplementary Note 17. Cellular viability assays

Supplementary Note 18. Anti-fouling of microswimmer

Supplementary Note 19. Microswimmer migration together with human cells

Supplementary Note 20. ZIF-8 coated microswimmers

Supplementary Note 1. Microswimmer fabrication procedure

The core/shell p/n junction silicon nanowire microswimmer was fabricated by previously reported vapor-liquid-solid (VLS) growth methods.[1] The <111> oriented silicon wafer with 150 nm thermal oxidized SiO_2 was used as substrate. The wafer was firstly cleaned in DI water, acetone and isopropanol for 5 min sequentially by sonication and blew dry with nitrogen gas. Then the wafer was transferred into fresh piranha solution (98% H_2SO_4:50% H_2O_2, 7:3, v/v) for 30 min to remove organic residues. After thoroughly rinsed with DI water, holes array with a diameter of ~1.5 μm and 4 μm pitch on the substrate was photolithographically patterned with G-line photoresist (OCG 825 35CS, FujiFilm, Inc.). Reactive-ion etch w μas used to etch through the SiO_2 layer with the photoresist as etching mask. 20 nm platinum was deposited into the holes by magnetron sputtering and lift-off process. Silicon nanowires with around 10 μm in length were grown in a CVD furnace for 10-30 min at 900 ^oC and ambient pressure with 350-550 sccm H_2/Ar carrier gas (1:10, v/v) and 10-30 sccm of carrier gas bubbled through a liquid $SiCl_4$ source at 2 °C. After the nanowire growth, the Pt on the silicon nanowire was removed by fresh aqua regia solution for 5 hours. To further clean the silicon nanowire surface, 100 nm of dry thermal oxide was thermally grown on the nanowire at 1000 °C in O_2 for 30 min and removed with 5:1 buffered HF (BHF). After BHF etching, silicon nanowires were immersed in aqua regia solution for another 5 hours and followed by BHF treatment. After this process, silicon nanowire array with a uniform diameter and clean surface can be obtained. Thermal oxidation and BHF etching can be further applied to reduce the nanowire diameter.

To form the p/n junction, the n^+-Si shell can be produced by gas phase thermal diffusion method. Typically, a silicon handle wafer was spin-coated with As-354 Arsenic dopant (Filmtronics, Inc.) at 2000 rpm for 30 s and then baked at 200 ^oC on a hot plate for 10 min. The handle wafer was used as Arsenic source for doping. The Si nanowire array sample was placed face-to-face beneath the handle wafer with 400 μm spacers in between and then annealed at 900 ℃ under vacuum with 55 sccm H_2/Ar gas (1:10, v/v) flow for 2-3 min. After annealing, the sample was treated by 5:1 BHF for 20 s to remove the surface oxide. 0.5 nm Platinum nanoparticles were deposited on the surface of silicon nanowires as electrocatalyst by magnetron sputtering with a customized tilted rotary stage.

Supplementary Note 2. Polystyrene grafting and sulfonating procedure

The as-obtained silicon array was hydroxylated by immersed in fresh piranha solution for 10 min and then rinsed by de-ionized water and isopropyl alcohol. Then it was transferred into a covered glass container and heated on a hotplate at 80 ^{o}C, 50 μL 3-Bromopropyltrichlorosilane (96%, Fluorochem Ltd) was dropped into the container near the silicon array. After 20 min initiator anchoring, the wafer was rinsed with methanol and post-baked on a hotplate at 110 ^{o}C for 1 hour. Then 5 mL anhydrous anisole (99.7%, Sigma-Aldrich Ltd.), 5 mL anhydrous styrene (Sigma-Aldrich Ltd.), 25 μL 1,1,4,7,7-Pentamethyldiethylenetriamine (J&K scientific Ltd, 98%) and 17.5 μL Ethyl 2-bromoisobutyrate (J&K scientific Ltd., 98%) were mixed in a 50 mL round bottle flask. The solution was purged with N_2 gas for 10 min to remove all dissolved oxygen. The sample was immersed in the solution and 60 mg L-Ascorbic acid and 2.7 mg $CuBr_2$ was added. The monomer polymerization reaction was allowed to proceed on the silicon surface in nitrogen for 11 hours and can be quenched by blowing air into the flask. The sample was cleaned by rinsing with deionized water and anisole thoroughly afterward.

To get the ionic conductive polymer[2], 10 mL dichloroethane (99%) and 2 mL acetic anhydride (98%, Sigma-Aldrich Ltd.) were mixed in ice bath. Then 0.72 mL sulfuric acid (98%, AR) was slowly added and a clear solution of acetyl sulfate was obtained. The silicon nanowire array coated with polystyrene brush was immersed in the acetyl sulfate solution at 60 ^{o}C. The reaction was stopped after 5 hours by rinsing with methanol. Finally, the sulfonated polymer was rinsed with DI water and neutralized in 0.5 M $NaHCO_3$ aqueous solution for 10 min.

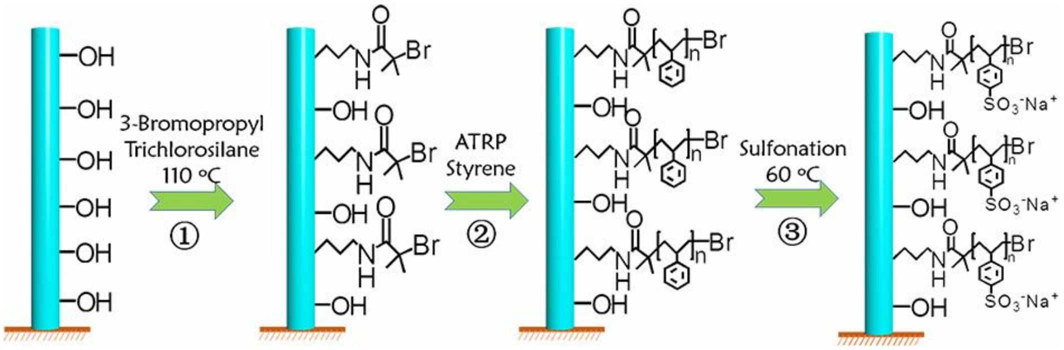

Supplementary Figure 1. Sulfonated poly(styrene) (SPS) grafting process.

Supplementary Note 3. SPS thickness characterization

To characterize the polymer thickness, LUDOX TM-50 colloidal silica (Sigma-Aldrich Ltd.) was chosen as a standard sample to measure the size distribution before and after coating SPS by dynamic light scattering (Nanotrac Wave II in Microtrac). Firstly, LUDOX TM-50 colloidal silica was diluted to 0.5%wt using 0.01 M KCl. The size of the pristine colloidal were measured firstly. The silica colloidal was then centrifuged down, 8000 rpm 10 min three times, and re-dispersed into anhydrous ethanol for the SPS coating. The coating process was the same as mentioned above and each process products were centrifuged down by 8000 rpm for 10 min three times. The size of the colloidal silica after SPS coating can be measured, and the polymer thickness was calculated by subtracting the diameter of the pristine particle (Supplementary Figure 2).

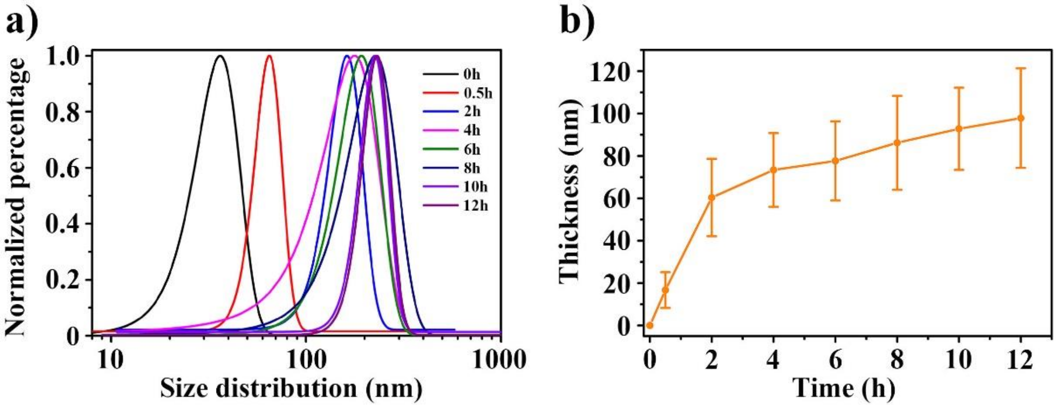

Supplementary Figure 2. The thickness of polymer coating vs. reaction time measured by DLS in aqueous solution. **(a)** Normalized size distribution of colloidal silica nanoparticles with different reaction time. **(b)** The plot of polymer thickness dependence over reaction time. Note, the size of nanoparticle has been subtracted.

Supplementary Note 4. Zeta potential measurement

The silicon nanowires made by electroless etching was synthesized using the method reported previously.[3] The as-obtained silicon nanowires were transferred into water and break into small segment by ultrasound for 10 h. Then the nanowires were transferred into DI water and washed by DI water for 3 times. The coating process of the silicon nanowires were the same as mentioned before.

The synthesized silicon nanowires with and without SPS coating were further rinsed by DI water before Zeta potential measurement. Then a pH buffer solution was prepared containing 0.035 mM sodium acetate and 0.015 mM acetic acid and the final PH was adjusted by HCl and NaOH to ~5.4. After that the nanowires were dispersed into the buffer solution for Zeta potential measurement. 1 M salt (NaCl, KCl, KIO_3) aqueous solution with pH buffer (pH=5.4) was prepared and the nanowires containing the solution was mixed with 1 M salt aqueous solution to obtain different ionic strength. The pH of the sample soltuion was measured by pH meter (Mettler Toledo SevenCompact ph/Ion benchtop meter) before putting into the cuvette to make sure the pH was around 5.4. The zeta potential of silicon nanowires without and with SPS coating under different salt concentrations were measured by Malvern Nanosizer (Zetasizer Nano ZS, Malvern Panalytical Ltd.) and ZetaView PMX120 Nanoparticle Tracking Analyzer (NTA, Particle Metrix Inc.) separately. The result was presented in Supplementary Figure 3, in which 3 different salt (NaCl, KCl, KIO_3) were tested separately.

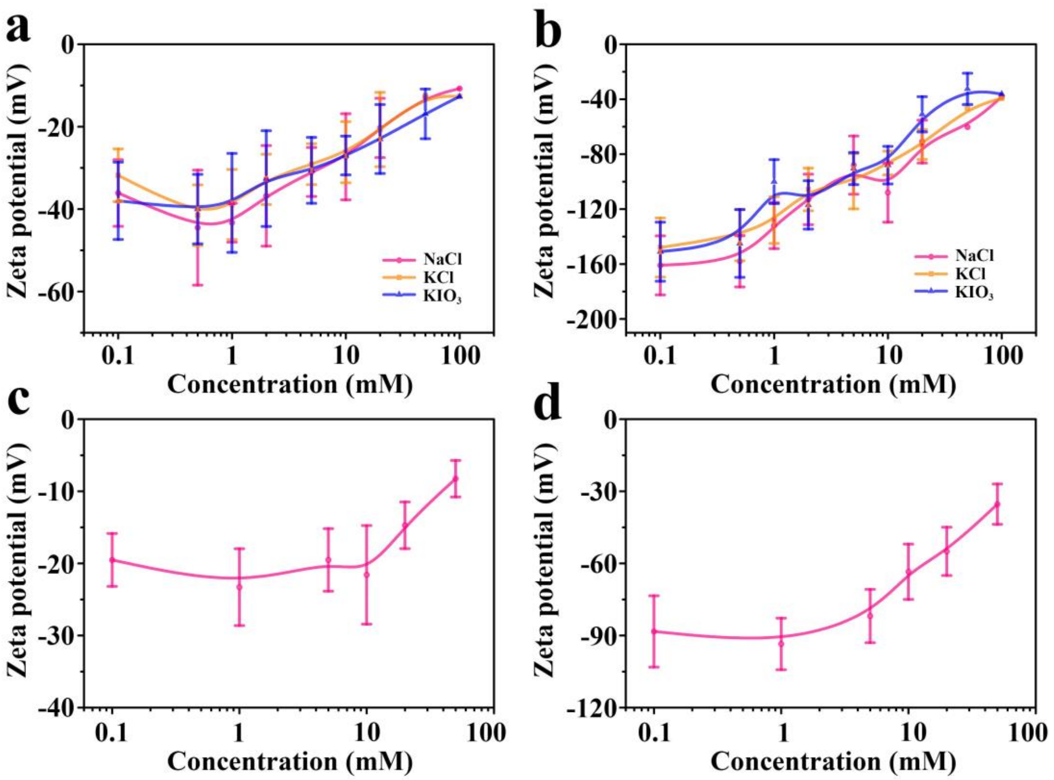

Supplementary Figure 3. The zeta potential of silicon nanowires **(a) (c)** without and **(b) (d)** with SPS coating in different salt concentrations (, and correspond to NaCl, KCl and KIO_3 respectively). (a)(b) were obtained from Malvern Nanosizer, (c)(d) were obtained from ZetaView PMX120 Nanoparticle Tracking Analyzer.

Supplementary Note 5. Electrochemistry characterization: RDE voltammetry

To investigating whether the diffusion is limited by the polymer coating, the RDE voltammetry was used to determine the diffusion coefficient before and after coating. All tests were conducted on Electrochemical Analyzer (CHI660E, CH Instruments, Inc.) using a standard three-electrode set-up with a homemade silicon disk working electrode (silicon sample stick on a platinum disk working electrode), while an Ag/AgCl reference and a platinum wire are used as the reference electrode and the counter electrode. To make the silicon disk working electrode, a high-doped n-type silicon wafer (resistivity ~0.0014 Ω·cm) loaded with platinum catalysts was glued to a platinum work disk electrode by silver paste. The silver paste and platinum was further sealed with nail polish. The RDE test was done in electrolyte solution with 2 mM benzoquinone and 1 M KCl at scanning range from -0.5 V to 0.1 V vs. Ag/AgCl and scanning rate at 0.01 $V \cdot s^{-1}$.

RDE voltammograms were analyzed via Levich plot and yielded the corresponding diffusion coefficient D by Levich equation $I_{Lim} = 0.62nFAD^{2/3}\nu^{-1/6}\omega^{1/2}c_0$, where n is the number of transferred electrons per redox reaction, F is Faraday's constant, A is the area of the electrode, ν is the viscosity of solution, ω is the rotation speed, c_0 is the bulk concentration of benzoquinone.

Supplementary Note 6. Microswimmer migration measurement procedure and data analysis

A customized glass slide with a 16 mm × 25 mm × 1 mm rectangle reservoir and array of numbered markers compose of 20nm thick titanium was prepared for this test. Two pairs of 2-pole gold electrodes (15 mm × 4 mm) with a 100 μm spacing was also patterned into the reservoir to in-situ measure the electrical conductivity of the media. This customized glass slide was thoroughly cleaned by solvent and O_2 plasma treated for 1 min before the microswimmer test. A series of standard NaCl solutions with known concentration (0~200 mM) were utilized to calibrate each slide before use.

The silicon nanowire microswimmers were scratched off from the substrate by a razor blade and dispersed into the mixed solution with 20 mM H_2Q and 10 mM BQ. The solution with microswimmers was then transferred into the reservoir of the testing slide for migration measurement. The chopped illumination was achieved by the ND filter. The conductivity of the solution was modulated by adding the BQ/H_2Q solution with high concentration NaCl. The reading from the electrical conductivity meter would be stabilized when equilibrium has been reached. The solution with different benzoquinone concentration was achieved similarly.

The speed of microswimmer was calculated by using in-house Matlab code. The position information of the microswimmer was captured frame by frame, and the speed was calculated. The consecutive frame by frame speed would be influenced by the Brownian motion significantly due to the short time interval, which shows a high speed deviation. For a typical video with 30 frames per second, the frame by frame speed deviation is around 1~2 $\mu m \cdot s^{-1}$, which is presented in Supplementary Figure 3a in the main text. In order to obtain high precision migration speed, over 200 speed points were used to calculate the average speed ($\bar{v}$) and the standard deviation (σ_v). And averaged speeds were calculated as confidence interval $v = \bar{v} \pm \frac{z\sigma_v}{\sqrt{N}}$ where z is 1.96 (95% confidence interval) and N is the number of the speed points. On the other hand, the Brownian motion under dark condition (< 1 $mW \cdot cm^{-2}$) was utilized as the background speed of microswimmer, which was subtracted to reflect the directional migration of the microswimmer. The averaged Brownian motion is calculated by equation $v = \bar{v} \pm \frac{z\sigma_v}{\sqrt{N}}$, and the value is around $0.31 \pm 0.02\ \mu m \cdot s^{-1}$with 95% confidence interval. For example, a typical calculated slow migration ($0.94 \pm 0.07\ \mu m \cdot s^{-1}$) and the speed is reported as $(0.94 - 0.31) \pm \sqrt{0.02^2 + 0.07^2})\ \mu m \cdot s^{-1}$, which reflects the slow directional migration of the microswimmer ($0.63 \pm 0.07\ \mu m \cdot s^{-1}$).

Supplementary Note 7. Formula deduction

Similar to a short-circuited galvanic cell proposed by Paxton et al[4], our microswimmer can be regarded as a short-circuited solar cell connected with two resistor, solution conductance and surface conductance (Figure 1d).

At low Reynolds number, the propulsion force (F_{prop}) of microswimmer is balanced by the viscous drag force[5] (F_{drag}) from the solution, as shown in Equation S1:

$$F_{prop} = F_{DL} + F_{sps} = F_{drag} = \frac{4\pi\eta L}{ln\left(\frac{2L}{r}\right)-0.72}U \quad \text{(S1)}$$

Where η is the dynamic viscosity of solution, L is the length of the cylinder rod, r is the radius of microswimmer, and U is the swimmer speed.

The propulsion force was contributed from both the microswimmer surface (F_{DL}) as well as polyelectrolyte coating (F_{sps}). F_{DL} comes from the double layer outside the SPS layer:

$$F_{DL} = Q_{DL} \times E \quad \text{(S2)}$$

while the net charge in the double layer Q_{DL} could be estimated as:

$$Q_{DL} = 4\pi rL\sqrt{2\varepsilon\varepsilon_0 cRT}\,\sinh\frac{zF\zeta}{2RT} \quad \text{(S3)}$$

ε_0 is permittivity of free space, is ε dielectric constant of solution, c is the bulk electrolyte concentration, z is the charge of the ion in the bulk solution, ζ is the zeta potential of the microswimmer.

F_{sps} can be described as:

$$F_{sps} = Q_{sps} \times E \quad \text{(S4)}$$

Q_{sps} is the net charge of the SPS layer contribute from Na^+ and can be described as follows:

$$Q_{sps} = \frac{2\pi rLK^{\sigma}}{\mu} \quad \text{(S5)}$$

K^{σ} is sheet conductivity of the SPS layer, μ is the ion mobility of Na^+ in SPS layer.

The electric field (E) generated by the ionic current (I_c) could be estimated by Ohm's law:

$$E = \frac{I_c}{d}R = \frac{2I_c}{L}R \quad \text{(S6)}$$

where d is the effective distance between the anode and cathode and R is the total resistance. Numerical calculation is used to calculate the shape related constant β as:

$$R_{solution} = \frac{1}{\beta K^L} \quad \text{(S7)}$$

where $R_{solution}$ is the effective resistance from anode to cathode through the solution. A 3D core-shell model was built by the commercial COMSOL Multiphysics package as we previous reported[1]. $R_{solution}$ under different solution conductivity and different geometry can be calculated by the electric current module through applying a certain voltage between the two electrodes. As the resistance of SPS layer (R_{sps}) can be expressed as:

$$R_{sps} = \frac{L}{4\pi r K^{\sigma}} \tag{S8}$$

the total resistance (R) is the parallel resistance of $R_{solution}$, R_{DL} and R_{sps}. Since the R_{DL} is much larger than R_{sps}, its contribution can be igored as:

$$\frac{1}{R} = \frac{1}{R_{solution}} + \frac{1}{R_{sps}} + \frac{1}{R_{DL}} \approx \frac{1}{R_{solution}} + \frac{1}{R_{sps}} \tag{S9}$$

From Equation S1-S9, we can obtain:

$$U = \frac{ln\left(\frac{2L}{r}\right) - 0.72}{2\pi\eta} \times \left(4\pi r L\sqrt{2\varepsilon\varepsilon_0 cRT}\sinh\frac{zF\zeta}{2RT} + \frac{2\pi r K^{\sigma}}{\mu}\right) \times \frac{I_c}{L\beta K^L + 4\pi r K^{\sigma}} \tag{S10}$$

Since the polyelectrolyte coating will dominate the ion conductivity in the SPS coated microswimmer, the U can be simplified as:

$$U = \frac{ln\left(\frac{2L}{r}\right) - 0.72}{2\pi\eta} \times \left(4\pi r L\sqrt{2\varepsilon\varepsilon_0 cRT}\sinh\frac{zF\zeta}{2RT} + \frac{2\pi r K^{\sigma}}{\mu}\right) \times \frac{I_c}{L\beta K^L + 4\pi r K^{\sigma}}$$

$$\approx \frac{ln\left(\frac{2L}{r}\right) - 0.72}{2\pi\eta} \times \frac{2\pi r K^{\sigma}}{\mu} \times \frac{I_c}{L\beta K^L + 4\pi r K^{\sigma}} = \frac{ln\left(\frac{2L}{r}\right) - 0.72}{4\pi\eta\mu} \times I_c \times \frac{K^{\sigma}}{\frac{L\beta}{4\pi r}K^L + K^{\sigma}} \tag{S11}$$

To describe the importance of surface conductivity, the Dukhin number was firstly introduced by Lyklema[5]:

$$Du = \frac{K^{\sigma}}{aK^L} \tag{S12}$$

It is a dimensionless ratio of the surface conductivity K^{σ} to the fluid bulk conductivity K^L multiplied by the characteristic length of the particle "a". In the case of our microswimmer, the geometry factor "a" can be defined as:

$$a = \frac{L\beta}{4\pi r} \tag{S13}$$

"a" is neither simply proportional to the nanowire's length nor reversely proportional to the radius, because β is also related to the geometry. Combining Equation S11-S13, the speed equation could be simplified as:

$$U = \frac{ln\left(\frac{2L}{r}\right) - 0.72}{4\pi\eta\mu} \times I_c \times \frac{K^{\sigma}}{aK^L + K^{\sigma}} \tag{S14}$$

$$U_0 = \frac{ln\left(\frac{2L}{r}\right) - 0.72}{4\pi\eta\mu} \times I_c \times \frac{K^{\sigma}}{aK^L + K^{\sigma}}\Bigg|_{\mathrm{aK^L \ll K^{\sigma}}} \approx \frac{ln\left(\frac{2L}{r}\right) - 0.72}{4\pi\eta\mu} \times I_c \tag{S15}$$

$$U = U_0 \times \frac{K^{\sigma}}{aK^L + K^{\sigma}} = U_0 \times \frac{Du}{1 + Du} \tag{S16}$$

$$U = U_0 \times \frac{K^{\sigma}}{aK^L + K^{\sigma}} = U_0 \times \frac{K^{\sigma}}{a\Lambda_m C + K^{\sigma}} \tag{S17}$$

By the definition of EI_{50}: $\frac{1}{2}U_0 = U_0 \times \frac{K^{\sigma}}{a\Lambda_m EI_{50} + K^{\sigma}} \Rightarrow EI_{50} = \frac{K^{\sigma}}{a\Lambda_m}$ (S18)

Λ_m is the molar conductivity of NaCl. A standard impedance measurement is utilized to measure the polymer conductivity on a glass slide, and the yielded polymer surface conductivity K^{σ} is

about$(2.18 \pm 0.45) \times 10^{-8}$ S·□, with the error contributed from data fitting error, equipment and electrodes size uncertainty.

As for bare microswimmer, the Dukhin number was calculated by using Equation S19, S20 developed by Lyklema [6]:

$$Du = \frac{2\left(1+\frac{3m}{z^2}\right)}{ak}\left(\cosh\frac{zF\zeta}{2RT} - 1\right) \quad \text{(S19)}$$

$$m = \frac{2\varepsilon_r\varepsilon_0 R^2 T^2}{3\eta F^2 D} \quad \text{(S20)}$$

Supplementary Note 8. Migration behavior of bare microswimmers on the SPS coated substrate

The glass substrate was coated with SPS using the method mentioned in Supplementary Note 2, and used as the substrate for analyzing the moving behavior of bare microswimmers. The contact angle of the substrate with water was measured for each step of SPS coating, as shown in Supplementary Figure 4, indicating the surface property change along the coating process. The volume of liquid was fixed at 1 μL by customized syringe pump system. As shown in Supplementary Figure 5, the ion tolerance of bare microswimmer on SPS coated glass substrate was not significantly higher than the ion tolerance of microswimmer on the glass substrate.

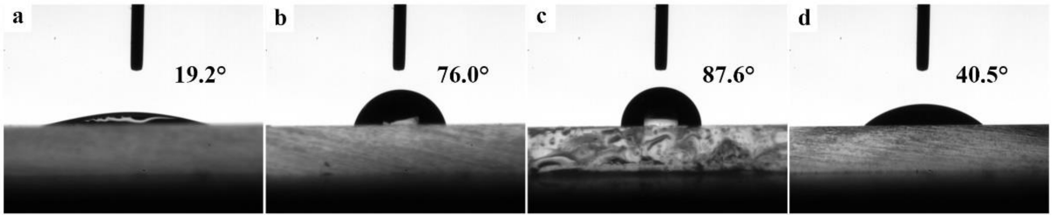

Supplementary Figure 4. The contact angle change of glass substrate in each step of SPS coating. **(a)** Hydroxylated glass substrate surface after immersed in a piranha solution. **(b)** Vapor phase coated glass substrate surface with initiator 3-Bromopropyltrichlorosilane. **(c)** Polystyrene-coated glass substrate surface. **(d)** Sulfonated polystyrene coated glass substrate surface.

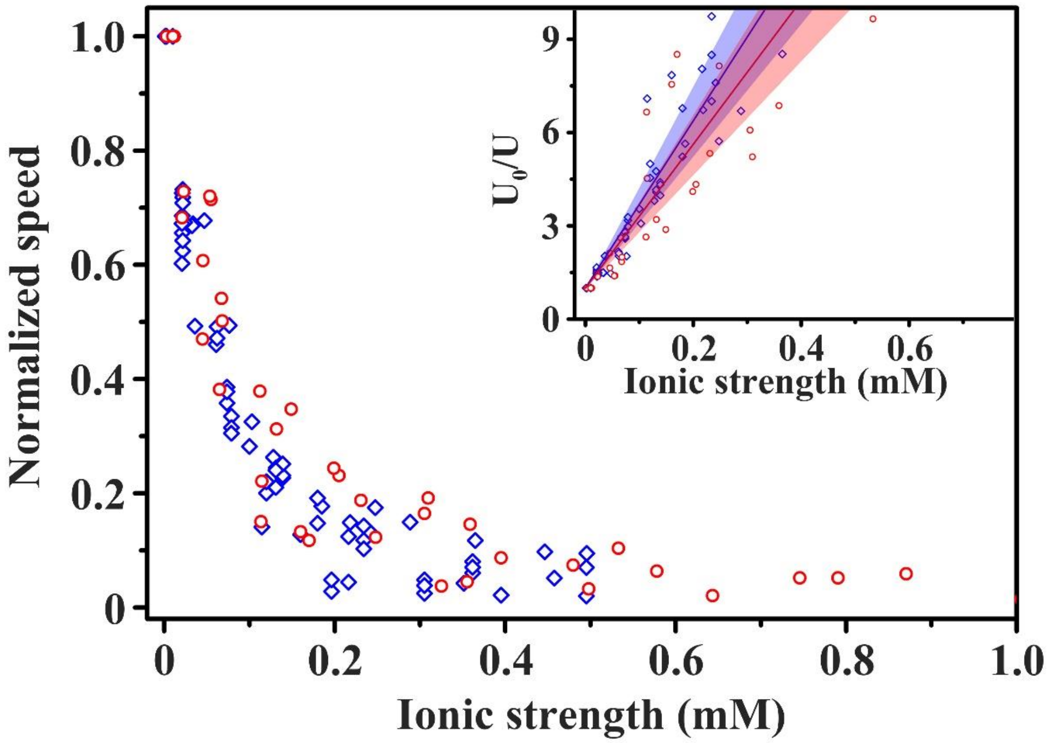

Supplementary Figure 5. The dependence of the normalized speed of the bare nanowire microswimmer on bare glass (blue) and SPS coated glass (red) to the solution ionic strength (number of counted microswimmers for each condition are 15 respectively). The inset shows the linear relationship between the normalized reciprocal speed and the electrolyte concentration as $\frac{U_0}{U} = 1 + \frac{1}{EI_{50}}C$. The solid lines and corresponding color bands indicate the fitting plots and the confidence interval of experimental data is at 95% confidence level. $EI_{50}^{\text{bare}} = 0.037 \pm 0.008$ mM and $EI_{50}^{\text{SPS substrate}} = 0.043 \pm 0.013$ mM.

Supplementary Note 9. Impedance measurement of SPS layer

A standard impedance measurement was utilized to measure the polymer conductivity on a glass slide. A glass slide was immersed in fresh piranha solution for 10min and then rinsed by DI water and IPA. Then using the same method to anchor the initiator and place the glass slide inside the reaction flask together with nanowire wafer. After obtaining a uniform SPS film, two electrodes (2 mm width with 2 mm gap distance) were patterned on the SPS coated glass slide. A piece of PDMS elastomer was gently pressed against the glass slide to hold a thin layer of water between the slide and the elastomer. 1 MΩ standard resistor was in series with the two electrodes and connected to the output of lock-in amplifier (SR830, Stanford research systems, Inc.) and measured at a frequency of 180 Hz. The partition voltage U_1 was also measured with the lock-in amplifier.

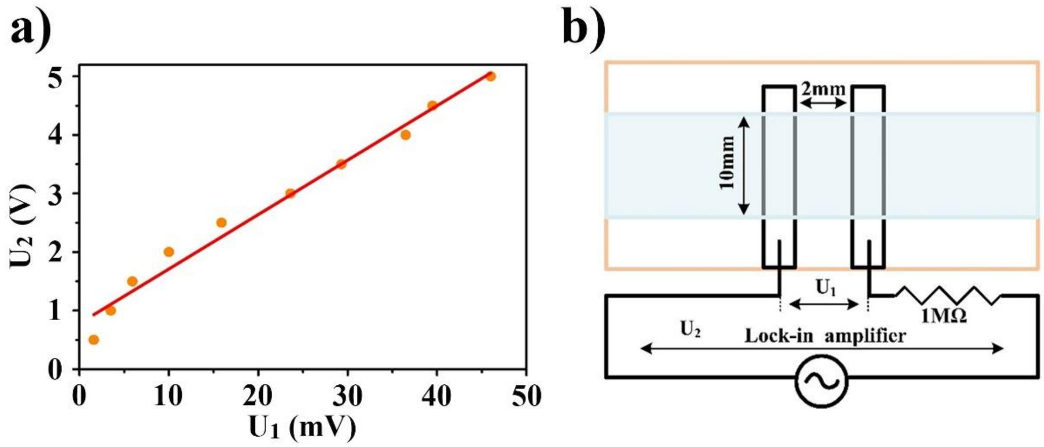

Supplementary Figure 6. SPS surface conductivity measurement using impedance measurement. **(a)** The partition voltage relationship between the sample and supply voltage. **(b)** Schematics of impedance measurement setup.

Supplementary Note 10. Ion tolerance improvement of SPS coated microswimmer ensemble

To compare the ion tolerance improvement of SPS coated microswimmer, more long SPS coated nanowire microswimmers were measured and grouped together with normalized speed (number of counted microswimmers, n=15). The ensemble data was shown in Supplementary Figure 7, where the ion tolerance was increased from $EI_{50}^{\text{bare}} = 0.037 \pm 0.008$ mM to $EI_{50}^{\text{SPS long}} = 0.232 \pm 0.064$ mM.

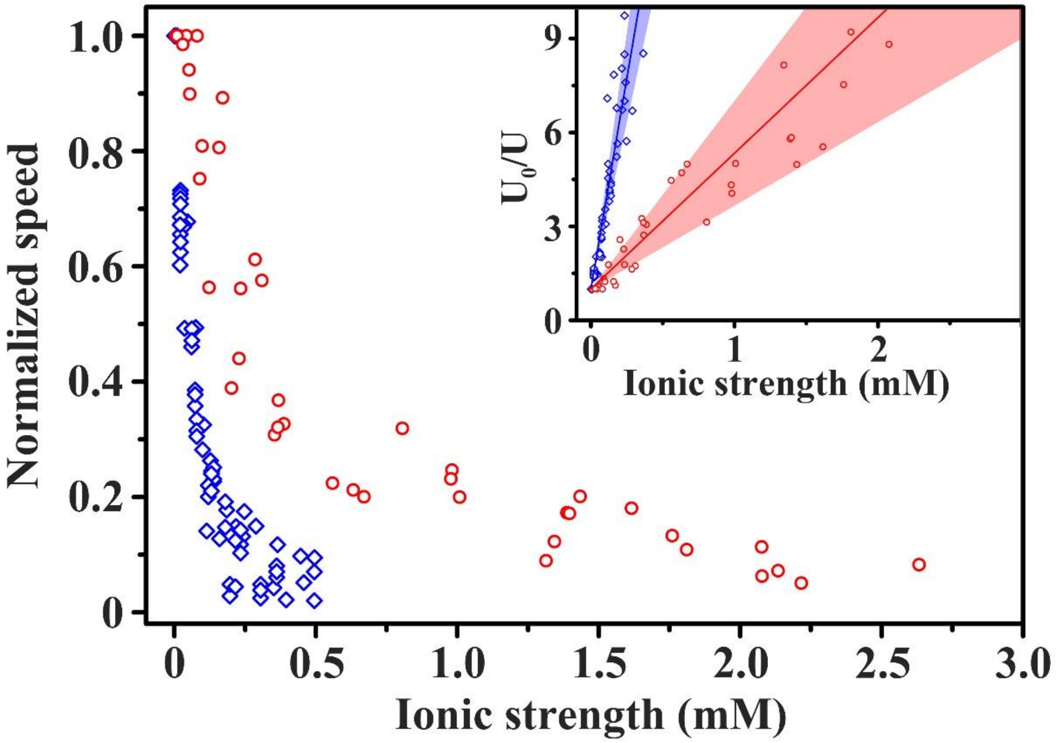

Supplementary Figure 7. The dependence of the normalized speed of the bare (blue) and the long SPS coated (red) microswimmer (~15 μm) to the solution ionic strength. The number of bare and SPS coated microswimmers is 15 respectively. The inset shows the linear relationship between the normalized reciprocal speed and the electrolyte concentration as $\frac{U_0}{U} = 1 + \frac{1}{EI_{50}}C$. The solid lines and corresponding color bands indicate the fitting plots and the confidence interval of experimental data is at 95% confidence level. $EI_{50}^{\text{bare,ensemble}} = 0.037 \pm 0.008$ mM and$EI_{50}^{\text{SPS,ensemble}} = 0.232 \pm 0.064$ mM.

Supplementary Note 11. Geometric factor of active bare and SPS coated microswimmer

Similar to microelectrode in pure water, due to the high impedance of the solution, the electrochemical reaction and the resulting electrical field is localized at the edge of the PN junction of the bare microswimmer at low ionic strength condition(Supplementary Figure 8a). At higher ionic strength, the solution impedance is lower and more nanowire surface is involved in the redox reaction (Supplementary Figure 8b). This scenario suggests that although the nanowire is long, only a small fraction of it can be counted for the microswimmer migration and this fraction increase its size upon higher electrolyte concentration.

For SPS coated swimmer, the SPS layer forms the high conductivity ion channel which makes the whole nanowire surface engaged in the reaction regardless of the ionic strength of the solution (Supplementary Figure 8c).

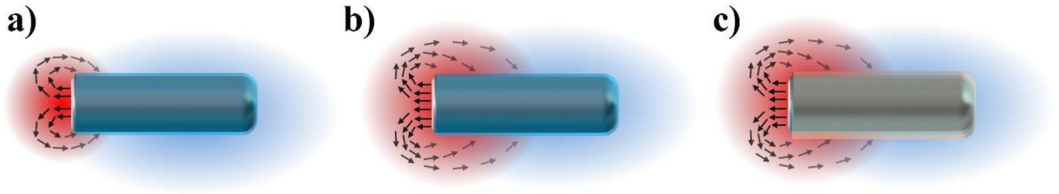

Supplementary Figure 8. Schematic diagram of the active area of microswimmer with and without SPS coating. **(a)** Bare microswimmer at low ionic strength condition. **(b)** Bare microswimmer at high ionic strength condition. **(c)** SPS coated microswimmer.

According to Equation 1 in the main text, $\frac{U}{U_0} = \frac{K^\sigma}{K^\sigma + aK^L}$, the geometric factor "$a$" can be calculated by fitting the slope of $\left(\frac{U_0}{U} - 1\right) \cdot K^\sigma$ with bulk solution conductivity (K_L), where the surface conductivity K^σ can be calculated by Equation S21 developed by Bikerman[6].

$$K^\sigma = \frac{4F^2 C z^2 D\left(1+\frac{3m}{z^2}\right)}{RT\kappa}\left(cosh\frac{zF\zeta}{2RT} - 1\right) \tag{S21}$$

As shown in Supplementary Figure 9a, the slope of bare microswimmer increased gradually as the solution conductivity increase, corresponding to larger active geometric size. On the other hand, SPS coated microswimmer showed a constant geometric size "a" within the tested range of solution conductivity (Supplementary Figure 9b). The fitted geometric factor of bare microswimmer and SPS coated microswimmer at different solution conductivity is shown in Supplementary Table 1.

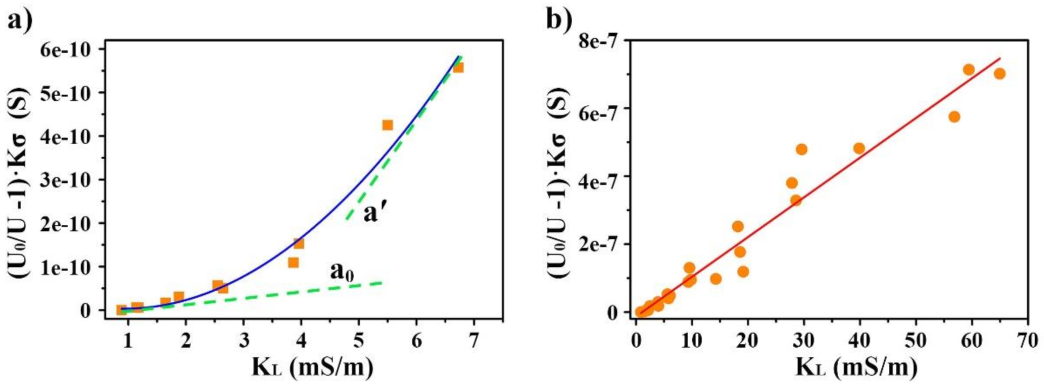

Supplementary Figure 9. Geometric factor fitting for **(a)** bare microswimmer and **(b)** SPS coated microswimmer. The geometric factor "a" changes significantly with different bulk conductivity for bare microswimmer while a constant "a" was observed for SPS coated microswimmer. The fitting geometric factor "a" for the SPS coated microswimmer is 1.2×10^{-5} m.

Supplementary Table 1. List of fitted geometric factor for the bare microswimmers with different geometric size. The bulk conductivity was fixed at 1×10^{-3} S·m^{-1}. The raw speed of the microswimmers can be found in Supplementary Note 14.

**Length (μm)**	**Diameter (μm)**	**Fitted geometric factor (m)**	
		**a_0 (K_L=1×10^{-3}S·m^{-1})**	**a' (K_L=7×10^{-3}S·m^{-1})**
2.3	0.4	2.9×10^{-8}	2.6×10^{-7}
3	0.4	3.2×10^{-8}	3.2×10^{-7}
14	0.6	1.6×10^{-8}	8.3×10^{-8}
15	0.8	4.3×10^{-8}	1.4×10^{-7}
15	0.6	2.2×10^{-8}	8.9×10^{-8}
28	0.6	6.5×10^{-8}	9.0×10^{-7}
30	0.6	5.2×10^{-8}	6.8×10^{-7}
30	0.6	3.7×10^{-8}	4.9×10^{-7}

With SPS coating, the surface conductivity was more than three orders of magnitude larger than bare microswimmer (Supplementary Figure 10a) which should in principle enhance the ion tolerance by three orders of magnitude. However, the total enhancement was the product of both surface conductivity enhancement and the geometric factor enhancement. While the geometric factor "a" increased significantly with the SPS coating, which largely canceled the enhancement from the surface conductivity (Supplementary Figure 10b), lower Dukhin number and less pronounced ion tolerance enhancement was observed (Supplementary Figure 10c).

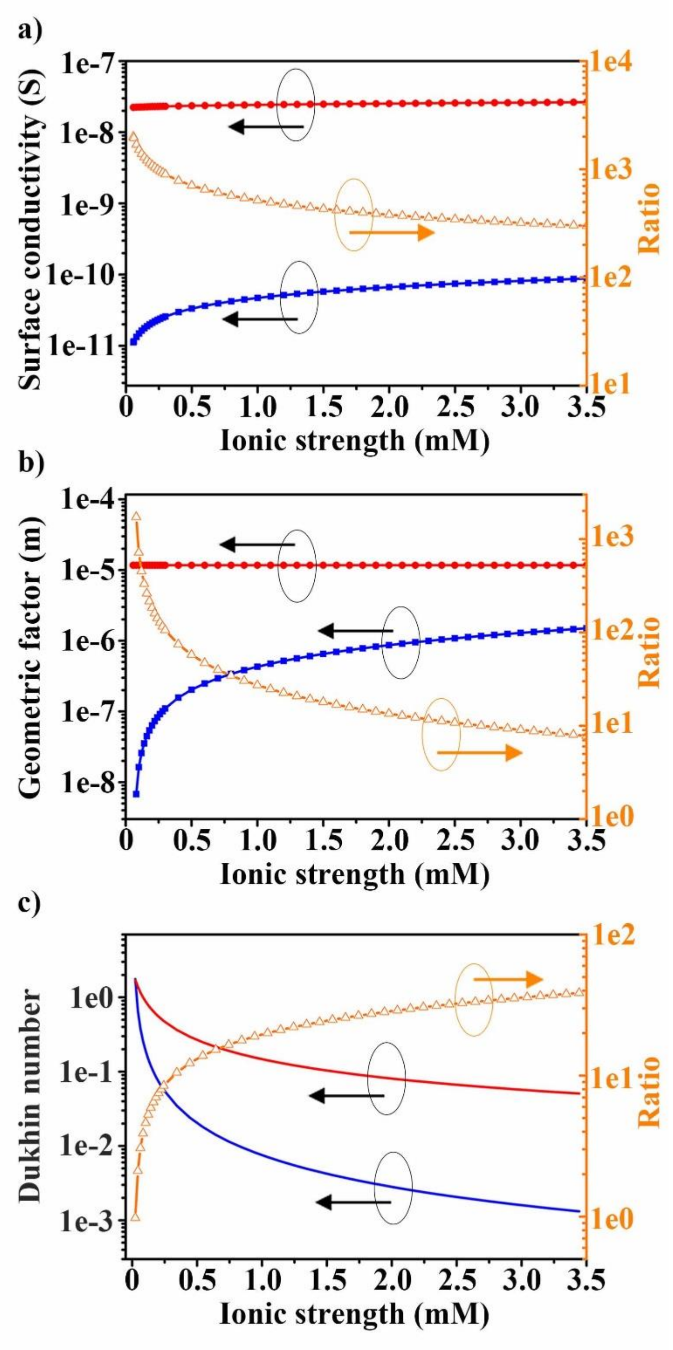

Supplementary Figure 10. The effect of SPS coating on the properties of 15 μm long nanowire microswimmer. **(a)** Surface conductivity **(b)** Geometric factor "a" and **(c)** Dukhin number of uncoated motor (blue) and SPS coated long motor (red) (black left y-axis). The yellow line indicates the corresponding ratios of SPS coated surface to the bare surface (orange right y-axis)

Supplementary Note 12. EOF velocity field simulation

Ion-induced charge distribution and flow speed were simulated using the commercial COMSOL Multiphysics package. 2D models were adapted from our previously reported silicon nanowire system. Hydroquinone/benzoquinone (H_2Q/BQ) system was taken as a representative system for numerical study. Cations (H^+) and anions (OH^-) are generated at anode and cathode surfaces, respectively and were further distributed by the diffusion, convection and migration of ions (Equation 22):

$$\nabla J_i = u\nabla c_i - D_i\nabla^2 c_i - \frac{z_i F D_i \nabla(c_i \nabla\emptyset)}{RT} \quad (22)$$

where J_i is the flux of ion i, u is the fluid velocity, F is the Faraday constant, ϕ is the electrostatic potential, R is the gas constant, T is the temperature and c_i, D_i, z_i are the concentration, diffusion coefficient, and charge of species i, respectively. The H^+ and OH^- will react to form H_2O quickly when they encounter each other.

The electric field E (E= -∇ϕ) in Equation S22 was calculated using the Poisson equation:

$$-\varepsilon_0\varepsilon_r\nabla^2\emptyset = \rho_e = F(z_+c_+ + z_-c_-) \quad (23)$$

where ε_0 is the vacuum permittivity and ε_r is the relative permittivity of water, $z_+ = +1$ and $z_- = -1$, ρ_e is the volumetric charge density, F is the Faraday constant and c_+ and c_- are the concentration of the H^+ and OH^-, respectively.

Electroosmotic boundary of the nanowire was set to -30 mV based on the measured zeta potential value (-30.09 mV). For the model with SPS coating, a 100 nm thick high ion conductive domain with 0.218 S/m conductivity was added on the nanowire surface. The steady ion flux on the anode and cathode ends are adopted based on the experimental photocurrent density.

Supplementary Table 2. Typical parameters used in the simulations. P-type silicon surface 90° relative to the axis of nanowire was chosen as a representative.

**Parameters**	**Meaning**	**Value**
D_1	H^+ diffusion coefficient	9.31e-5 [$cm^2 \cdot s^{-1}$]
D_2	OH^- diffusion coefficient	5.03e-5 [$cm^2 \cdot s^{-1}$]
Ic	Cathode photocurrent density	4.84 [$A \cdot m^{-2}$]
Ia	Anode photocurrent density	0.118 [$A \cdot m^{-2}$]
Ec	Cathode equilibrium potential	- 0.124 [V]
Ea	Anode equilibrium potential	0.34 [V]
L	Length of nanowire	10 [μm]
d	Diameter of nanowire	1 [μm]

Supplementary Table 3. List of parameters used in the proposed equations.

Parameters	Meaning	Value for Figure 3		Value for Figure 5		
		bare	SPS coated	short	medium	long
L	length of the cylinder rod (μm)	15	15	2	4.3	15
r	radius of microswimmer (μm)	0.3	0.3	0.2	0.2	0.3
ξ	zeta potential of microswimmer (mV)	-30.09	-160	-160		

Supplementary Note 13. Ion tolerance of EOF for different ions

Electroosmosis flow speed was measured on SPS coated substrate and the ion tolerance was tested with different ions. Briefly, a pair of gold electrodes was patterned on glass with and without SPS coating and the electrode separation is 2 mm. A customized PDMS flow cell with cover glass was bonded on the sample. Solutions of different electrolyte are injected from the incorporated tube inlet and + 0.5 V voltage (2635B Keithley Instruments) was applied on the two electrodes and 1 μm neutral PS sphere was used as the tracer. In all solution, 20 mM H_2Q and 10 mM BQ were added as redox shuttle. The electroosmotic flow (EOF) speed was calculated by using in-house Matlab code. For each video, the number of tracer particle counted is 30. As shown in Supplementary Figure 11, the SPS coating enhances the EOF ion tolerance similar to the microswimmer as described in the main text. On the other hand, the NaCl shows largest enhancement similar to KCl, while the KIO_3 shows smallest enhancement. One possible explanation can be related to the additional apparent zeta potential due to different interaction potential of ions to the polymer coating (Φ).[7] Since the SPS is negatively charged, the interaction of positively charged cation is greater than negatively charged anion, while the relative interaction strength of K^+, Na^+, Cl^- , and IO_3^- can be judged by their hydrodynamic radius, which is assumed as $\Phi_{Na^+} > \Phi_{K^+} > \Phi_{Cl^-} > \Phi_{IO_3^-}$. In this case, the KIO_3 has largest anion-cation interaction potential difference and the additional apparent zeta potential will be largest and can cancel the zeta potential quicker than NaCl which has smaller anion-cation interaction potential difference. It should be noted that this model[7] assumes low porosity polymer coating and no EOF penetration inside the polymer coating layers which is not the case for our experiment, while the predicted migration direction reversal are also not observed in our experiment. In our experiment, the low porosity/high density SPS layer coated microswimmer failed to show enhanced ion tolerance, which also suggested additional physics in this system. Further experimental and theoretical studies are needed to elucidate the distinct nature of ion species tolerance. However, since the dominate electrolyte for in-vivo conditions are NaCl and small amount of KCl, the dependence of other ions are less important from application point of view and not covered in this study.

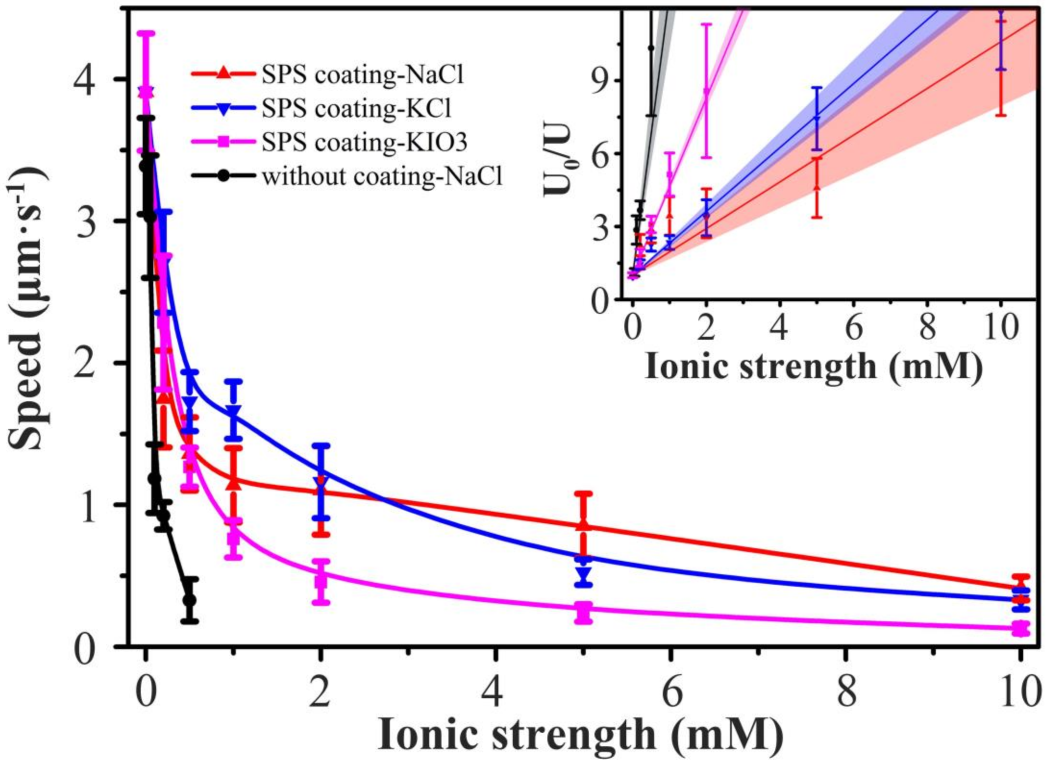

Supplementary Figure 11. The electroosmosis speed vs ionic strength of different electrolytes. Black (no coating, NaCl), red (SPS coated, NaCl), blue (SPS coated, KCl) and pink (SPS coated, KIO_3). The error bar is estimated with the standard deviation of multiple particle speed measurement (n=30). The inset shows the linear relationship between the normalized reciprocal speed and the electrolyte concentration as $\frac{U_0}{U} = 1 + \frac{1}{EI_{50}}C$. The solid lines and corresponding color bands indicate the fitting plots and the confidence interval of experimental data is at 95% confidence level. $EI_{50}^{\mathrm{bare}} = 0.086 \pm 0.039$ mM, $EI_{50}^{\mathrm{SPS\ NaCl}} = 1.041 \pm 0.514$ mM, $EI_{50}^{\mathrm{SPS\ KCl}} = 0.761 \pm 0.162$ mM and $EI_{50}^{\mathrm{SPS\ KIO_3}} = 0.275 \pm 0.050$ mM.

Supplementary Note 14. Enhanced ion tolerance with geometry optimization

The migration behavior was collected and presented together with the geometry size to analysis and test our proposed model. The migration behavior of bare microswimmer in electrolyte solution was shown in Supplementary Figure 12, corresponding geometric parameters and EI_{50} was presented in Supplementary Table 4. No significant deviation from Helmholtz-Smoluchowski behavior was observed. As for SPS coated microswimmer (Supplementary Figure 13 & Supplementary Table 5), the ion tolerance was enhanced with smaller geometric size.

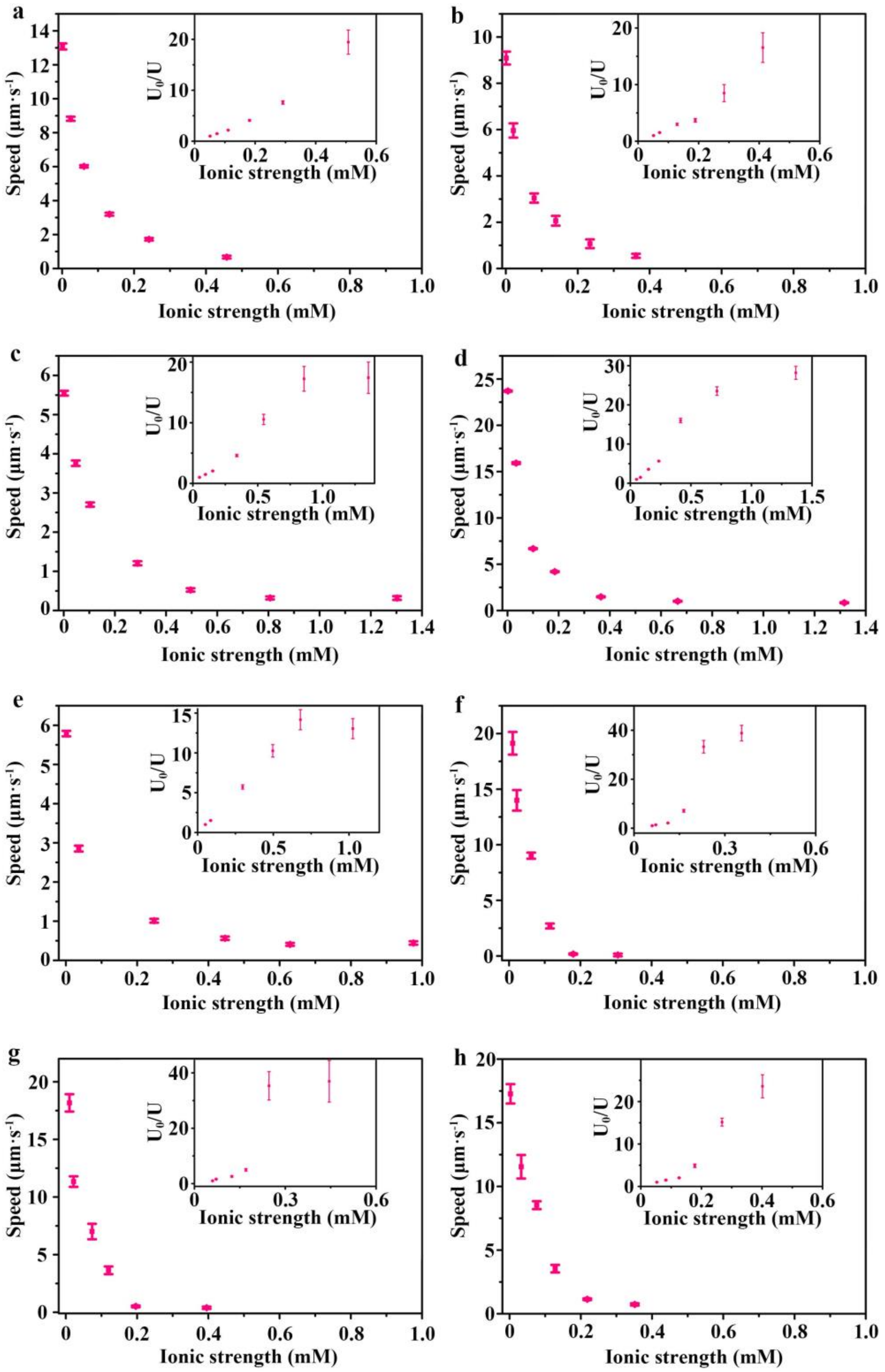

Supplementary Figure 12. The migration behavior of bare microswimmers **(a-h)** in different ionic strength solution. The insets are the corresponding inverted speed vs. ionic strength. The ionic strength was tuned by the concentration of NaCl.

Supplementary Table 4. Geometric parameters and EI_{50} of bare microswimmers **(a-h)**.

**Bare Microswimmer**	**Length (µm)**	**Diameter (µm)**	EI_{50} **(mM)**
a	2.3	0.4	0.025 ± 0.005
b	3	0.4	0.024 ± 0.006
c	14	0.6	0.048 ± 0.006
d	15	0.6	0.028 ± 0.009
e	15	0.6	0.047 ± 0.007
f	28	0.6	0.018 ± 0.008
g	30	0.6	0.028 ± 0.008
h	30	0.6	0.015 ± 0.006

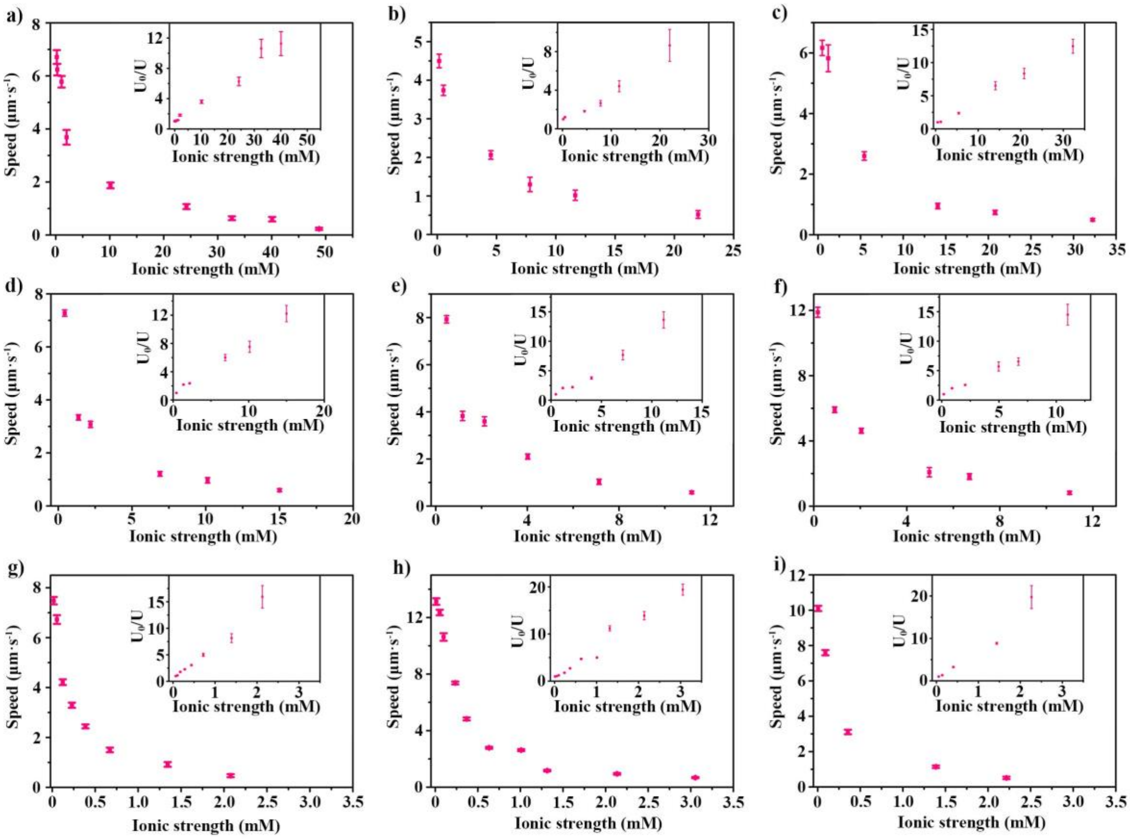

Supplementary Figure 13. The migration behavior of the SPS coated microswimmers **(a-i)** in different ionic strength solution. The insets are the corresponding inverted speed vs. ionic strength. The ionic strength was supported by the concentration of NaCl.

Supplementary Table 5. Geometric parameters and EI_{50} of SPS coated microswimmers **(a-i)**.

SPS Coated Microswimmer	Length (µm)	Diameter (µm)	Geometric factor "a" (µm)	EI_{50} (mM)
a	2	0.4	0.71	3.820 ± 0.294
b	2.1	0.4	1.05	3.521 ± 0.244
c	2.4	0.4	1.35	2.990 ± 0.259
d	4.3	0.4	2.32	1.436 ± 0.175
e	4.5	0.6	2.78	1.190 ± 0.129
f	6	0.6	3.75	0.852 ± 0.065
g	14	0.7	8.71	0.189 ± 0.017
h	15	0.6	10.79	0.218 ± 0.023
i	17	0.7	11.18	0.209 ± 0.015

Supplementary Note 15. Geometric factor "a" of SPS coated swimmer

The Dukhin number is a dimensionless quantity to compare the contribution from surface conductivity and the bulk solution. According to the definition of Dukhin number, the geometric size a is used for spherical colloidal particles, while the rod-like particles can be calculated from similar physical definition[8]. For active microswimmers with arbitrary geometry, the Du should be re-defined based on the physical meaning, which represent the ratio of the electrical conductance through the surface to the solution from the anode to the cathode. As mentioned in Supplementary Note 7, the shape related correction factor for R_{sps} is $\frac{4\pi r}{L}$ ($R_{sps} = \frac{L}{4\pi r K^{\sigma}}$, Equation S8) and the shape related correction factor for $R_{solution}$ is β ($R_{\text{solution}} = \frac{1}{\beta K^{L}}$, Equation S7). The contribution ratio depicted by Dukhin number is:

$$Du = \frac{R_{\text{solution}}}{R_{SPS}} = \frac{4\pi r K^{\sigma}}{L\beta K^{L}} \tag{24}$$

In order to reflect the shape related correction, the geometric factor "a" is defined as $a = \frac{L\beta}{4\pi r}$ (Equation 13). The results are shown as Figure 5a in the main text, in which the error bars of EI_{50} is the same with Supplementary Table 5, the error bars of geometric factor "a" reflects the size uncertainty obtained from the swimmers' tracking videos. The geometric size (length and diameter) of microswimmers were measured in aqueous medium under the optical microscope. Due to the resolution limit of the optical microscope, the uncertainty of the nanowire length and diameter obtained from the optical image was estimated by multiple measurements of the nanowire images. The upper and lower confidence limit of a is obtained by input the upper and lower boundary of length and diameter of the nanowires into the predicted geometric relation as shown in Figure 5a.

Supplementary Note 16. The ion tolerance enhancement of diffusiophoresis microswimmers

The TiO_2 and SiO_2 spheres were synthesized using the hydrothermal method. SiO_2 microspheres were synthesized using the similar process mentioned in zeta potential measurement with lower temperature (25 ℃). TiO_2 microspheres were synthesized following the previous paper.[9] The as-obtained spheres were dispersed into ethanol and then add to DI water dropwise to form a single layer of particles at the liquid-gas interface. Then clean silica wafer was used to scoop up the film and let dry on 80 ℃ hot plate to assemble to a monolayer on the substrate. 20 nm Pt was sputtered onto the film and the Janus microswimmers were obtained (Supplementary Figure 14). TiO_2/Pt or SiO_2/Pt Janus spheres were immersed into 1 $g \cdot mL^{-1}$ Poly(sodium 4-styrenesulfonate) (Sigma Aldrich) for 5 hours for polymer loading and then washed by DI water. TiO_2/Pt or SiO_2/Pt Janus spheres were transferred into 2%wt H_2O_2 solutions with different NaCl salt concentration for migration test under microscope. A halogen lamp equipped with 450 nm long-pass filter was utilized as illumination source. The videos were analyzed by Image J to obtain the tracking trajectories. The speed was obtained by analyzing 20 particles per condition. As shown in Supplementary Figure 15, The enhanced ion tolerance was observed in the SPS loaded microswimmers.

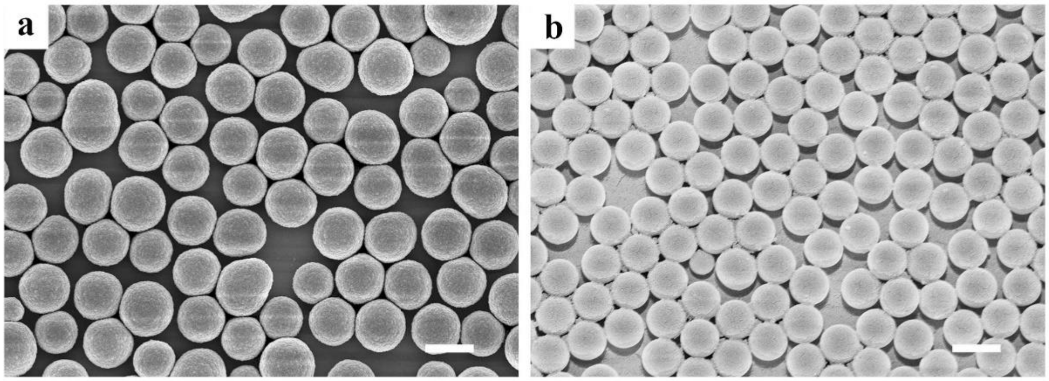

Supplementary Figure 14. SEM images of TiO_2/Pt **(a)** and SiO_2/Pt **(b)** Janus microspheres. The scale bar is 1 μm.

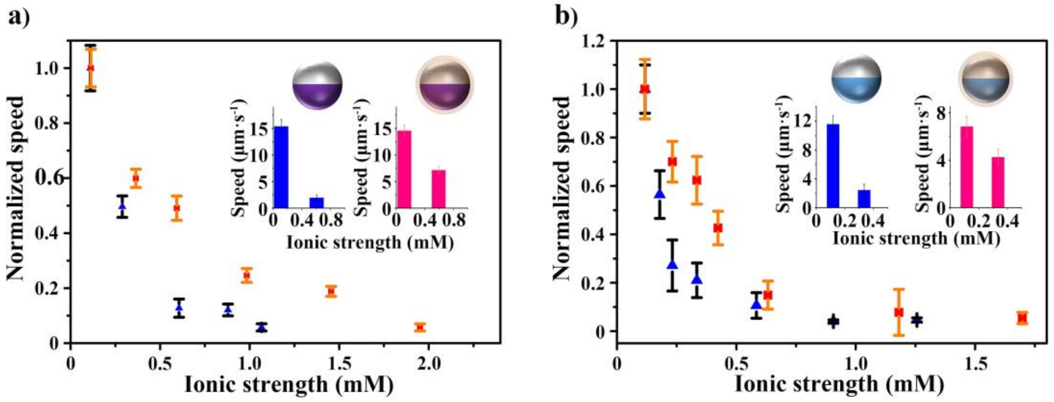

Supplementary Figure 15. Ion tolerance enhancement of diffusiophoresis microswimmers. **(a)** TiO_2/Pt Janus spheres in 2%wt H_2O_2 without (blue, black error bar) and with (red, orange error bar) PSS dipping. **(b)** SiO_2/Pt Janus spheres in 2%wt H_2O_2 without (blue, black error bar) and with (red, orange error bar) PSS dipping. The insets show the raw speed before and after adding the NaCl. (Number of the particles counted for each condition, n=20)

Supplementary Note 17. Cellular viability assays

To evaluate whether the H_2Q/BQ (20 mM/10 mM) solution used in this study is suitable for in-vivo application, the cytotoxicity of H_2Q/BQ was assessed with two different protocols. It is known that different cell lines could have different tolerance. Two cell lines NB4 and U2OS are selected for cytotoxicity evaluation, corresponding to adherent cells and suspension cell.

NB4 suspension cell

NB4 leukemia cell lines were cultured in RPMI1640 medium (Life Technologies) supplemented with 10% FBS and 1% penicillin-streptomycin solution (Life Technologies) (37 ℃, 5% CO_2). Cells were harvested by 3 repetitions of centrifugation (1000 g and 5 min) with 0.01 M ice-cold phosphate-buffered saline (PBS) at pH 7.4. After the treatment with H_2Q/BQ or H_2O_2, the cells were treated by Trypan blue exclusion staining. Prepare a 0.4% solution of trypan blue in PBS, add 0.1 mL of 0.4% trypan blue stock solution to 0.1 mL of cells. Mix well and wait for 1 min before loading onto a hemacytometer to examine the shape under the microscope.

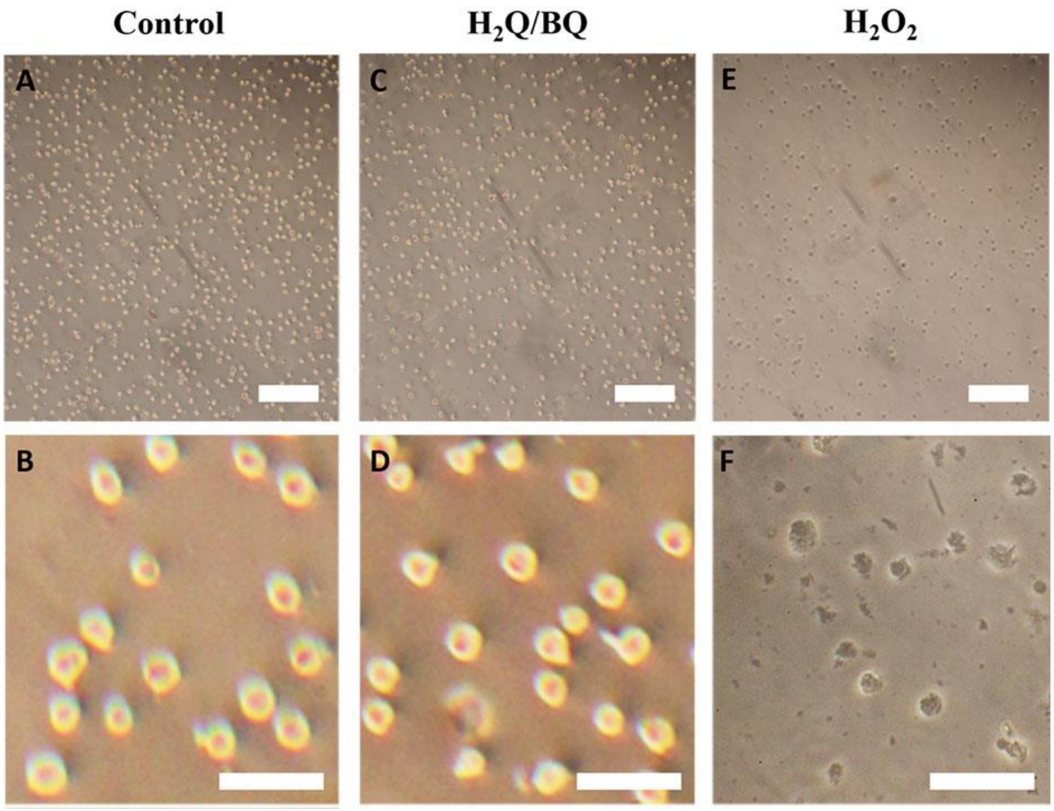

Supplementary Figure 16. The images of the NB4 cells cultured for 5.5 h with pure culture medium (**A** and **B**), H_2Q/BQ (20 mM/10 mM, **C** and **D**) and 0.1% H_2O_2 (**E** and **F**), respectively. The scale bar for A, C, E is 200 μm, and for B, D, F is 50 μm.

Because the absorption spectrum of H_2Q/BQ overlaps with the detecting reagent used for LD_{50} measurement, the cell proliferation after H_2Q/BQ treatment was characterized to evaluate the cytotoxicity of H_2Q/BQ to NB4 cell. The NB4 cells are treated with in H_2Q/BQ (20/10 mM) for a series of time (1 h, 2 h, 4 h, 6 h), then harvested by centrifugation and washed 3 times with PBS solution. The cell density of NB4 cells is counted and compared after 24 h re-culturing in RPMI1640 medium to evaluate the proliferation ability. In parallel, the NB4 cells without H_2Q/BQ treatment were also tested as a control sample. The cell density was counted by hemocytometer based on the standard protocol.[10] The errors are the standard deviation of the 3 independent replicates carried out for each set of cells. As shown in Supplementary Figure 17, compared to cells without H_2Q/BQ exposure, the cell proliferation is suppressed after H_2Q/BQ (20/10 mM) exposure, while the cell proliferation is still observed, which imply at least partial cell survive with the H_2Q/BQ treatment upto 6 hours of treatment.

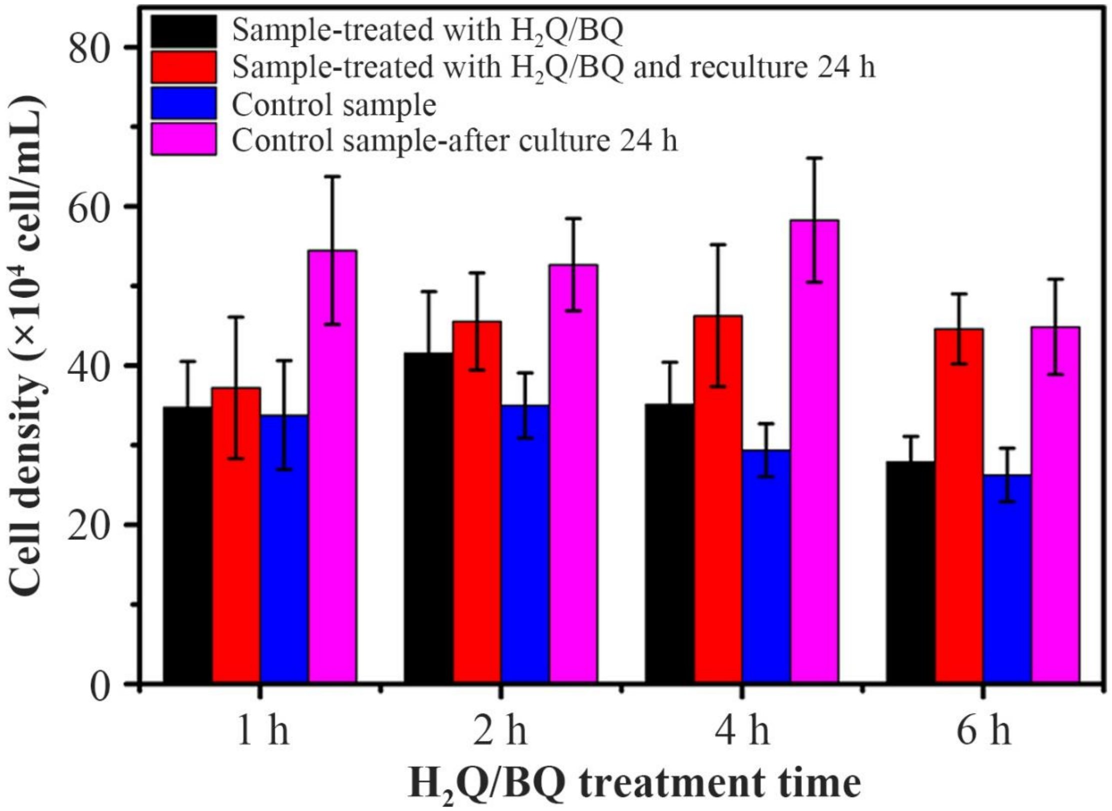

Supplementary Figure 17. The cell density comparison of NB4 cell treated with H_2Q/BQ and control sample without H_2Q/BQ treatment before and after re-culture 24 h.

LD_{50} measurement of U2OS cells for H_2Q/BQ. U2OS (ATCC) cells were cultured in DMEM medium supplemented with 10% fetal bovine serum, 100 U·mL^{-1} penicillin, and 100 μg·mL^{-1} streptomycin (Thermo Fisher Scientific). Cells were maintained in a humidified 37 °C incubator with 5% CO_2. For *LD_{50}* determination, U2OS cells were seeded into 96-well culture plates at the density of 2,000 cells per well in 100 μL medium. The compounds were added in triplicate to cells at indicated concentrations. After 48-hour incubation, the PrestoBlue® Cell Viability assay (Thermo Fisher Scientific) was used to determine the relative cellular viability by the detection of fluorescence (Excitation/Emission: 560/590 nm). The *LD_{50}* for H_2Q/BQ to U2OS cells is ~0.1mM, which implies that the U2OS cell cannot tolerant H_2Q/BQ for 48 hours.

In summary, the H_2Q/BQ shows lower toxicity than H_2O_2 and can partially support live cells upto 6 hours, while failed on 48 hours toxicity test. Another non-toxic redox active chemical is needed to overcome the cytotoxicity of nano/microswimmers.

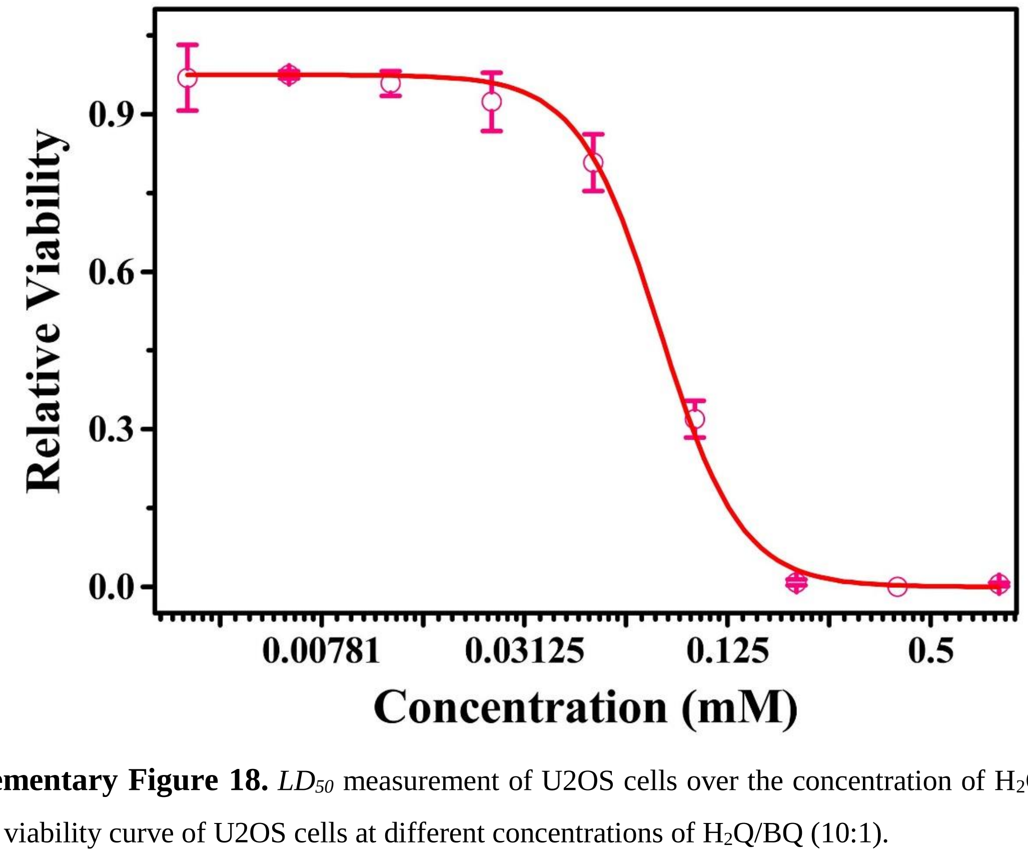

Supplementary Figure 18. *LD_{50}* measurement of U2OS cells over the concentration of H_2Q/BQ. The relative viability curve of U2OS cells at different concentrations of H_2Q/BQ (10:1).

Supplementary Note 18. Anti-fouling of microswimmer

The n^+ silicon wafer (0.0014 Ω·cm) was cleaned by a fresh piranha solution. Then 1 nm Pt was sputtered onto the surface of the wafer. Then SPS polymer was coated onto the wafer surface using the method mentioned above. The wafer was cut into 1 cm × 1 cm and the back side was polished by 1 M NaOH, then covered by In-Ga liquid metal to form an ohmic contact and glued on a platinum RDE electrode by silver paste as the working electrode. The electrode was further sealed with PMMA coating. The Pt wire was selected as a counter electrode, and Ag/AgCl electrode was used as a reference.

The I-V curve was measured in mixed solution with 10 mM benzoquinone in PBS solution with (v/v, Blood:PBS = 1:2) or without blood. The scanning range was set from -0.6 V to 0.2 V vs. Ag/AgCl at a scanning rate of 5 $mV \cdot s^{-1}$. Tafel plot was measured at suitable scanning range vs. Ag/AgCl and scanning rate at 5 $mV \cdot s^{-1}$. The linear range fitting allowed us to determine the exchange current density (j_0) and the kinetic constant (k^0) (Supplementary Figure 19).

It can be observed that upon contact with blood, the electrochemistry of both SPS coated and uncoated silicon electrode was slowed down due to the enzyme coating on the electrode which limited the migration speed of microswimmer. As shown in Supplementary Figure 20, the migration speed of SPS coated microswimmer in diluted PBS solution was decreased when human blood serum with the same ionic strength was added.

Comparatively, the SPS coated electrode was not affected as much as the bare silicon. However, further improvement was needed to prevent the biofouling in a biological fluid.

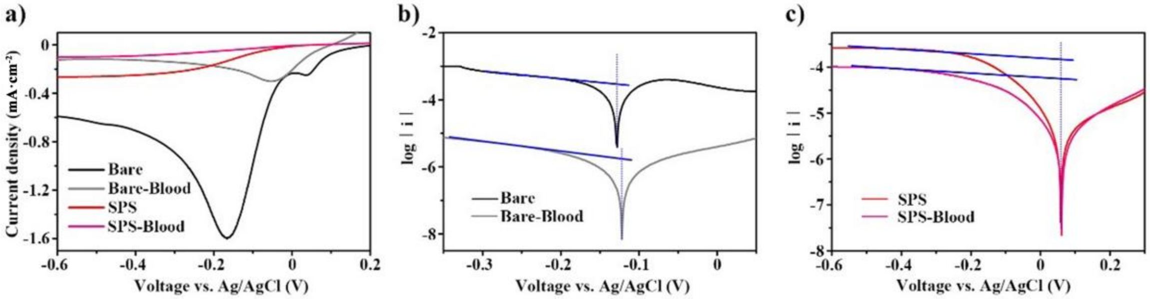

Supplementary Figure 19. Electrochemistry characterization of the silicon electrodes with and without SPS coating in human blood. **(a)** two-electrode I-V curve of bare and SPS coated n^+ silicon wafer in mixture solution of 10 mM BQ and 150 mM PBS with and without blood (Blood:PBS = 1:2). **(b)** Corresponding Tafel plot of bare n^+ silicon wafer in 10 mM BQ and 150 mM PBS solution without blood (black) and with blood (grey). The exchange current density i_0 was -2.88×10^{-4} mA·cm^{-2} (without blood) and -1.70×10^{-6} mA·cm^{-2} (with blood) respectively. The corresponding electrode reaction rate constants were 2.99×10^{-6} m·s^{-1} (without blood) and 1.76×10^{-8} m·s^{-1} (with blood). **c)** Corresponding Tafel plot of SPS coated n^+ silicon wafer in 10 mM BQ and 150 mM PBS solution without blood (red) and with blood (pink). The exchange current density i_0 was -1.48×10^{-4} mA·cm^{-2} (without blood) and -5.62×10^{-5} mA·cm^{-2} (with blood) respectively. The corresponding electrode reaction rate constants were 1.53×10^{-6} m·s^{-1} (without blood) and 5.83×10^{-7} m·s^{-1} (with blood). The scanning rate was 5 mV·s^{-1} for all the curves.

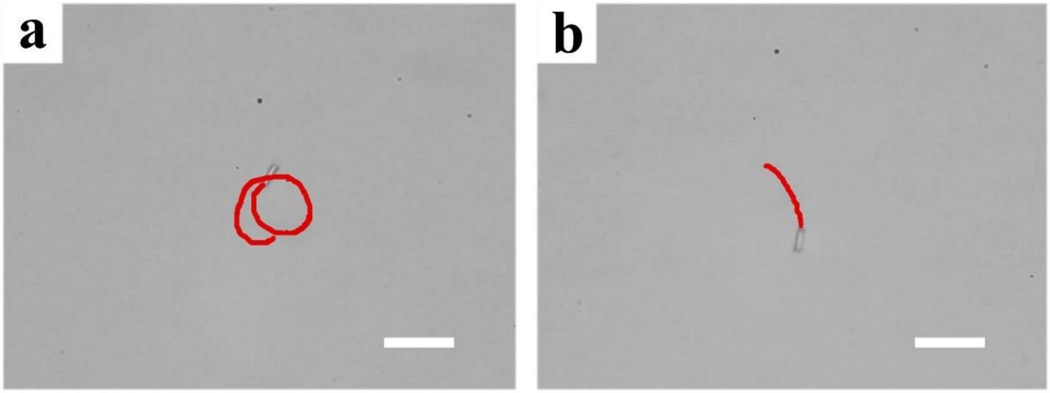

Supplementary Figure 20. The migration trajectory of the same SPS coated microswimmer **(a)** before and **(b)** after addition of human blood serum. The duration for both trajectories are 12 s and scale bars are 10 μm.

Supplementary Note 19. Microswimmer migration together with human cells

The acute promyelocytic leukemia NB4 cell (Creative Bioarray Inc.) was centrifuged down and dispersed into 40 mM PBS solution. The microswimmer was transferred into the 40 mM PBS solution. The PBS buffer solution containing NB4 cell was then added into the observation reservoir, and the video was recorded through the microscope.

The human blood was centrifuged at 5000 rpm for 5 min to separate the cell and serum before usage. The diluted human blood serum was prepared by mixing the serum with 10 mM PBS solution (v/v, 1:20). The human blood cells were washed three times by 150 mM PBS solution, and then were added to diluted human blood serum or diluted PBS solution (20 mM). The swimmers were transferred into the solution and loaded into 0.2×0.4 mm rectangle boro tubing (VITROCOM A fabrinet Company) for observation under the microscope (Nikon eclipse Ti2).

Supplementary Note 20. ZIF-8 coated microswimmers

The as-obtained silicon nanowire microswimmers were coated with Zeolitic imidazolate frameworks (ZIF-8). The conformal ZIF-8 coating was shown in Supplementary Figure 21a, and its migration with human blood cell in diluted PBS solution (Supplementary Figure 21b) was demonstrated.

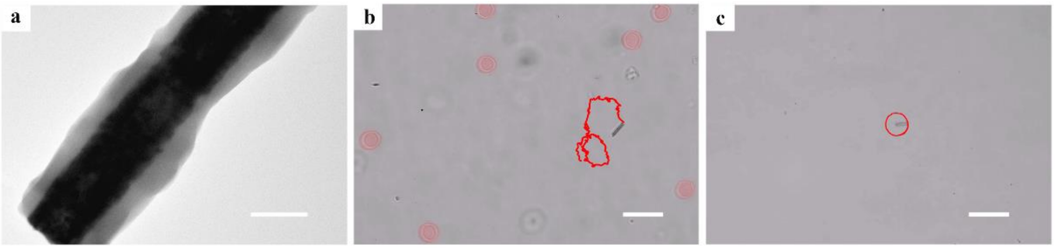

Supplementary Figure 21. TEM image and trajectory of ZIF-8 coated silicon nanowire microswimmer. **(a)** TEM image of ZIF-8 coated silicon nanowire. The scale bar is 100 nm. **(b)** The migration trajectory of ZIF-8 coated silicon nanowire microswimmer in diluted PBS solution mixed with human blood cells and 20 mM H_2Q, 10 mM BQ. The live human blood cells were red false-colored for clarity. The scale bar is 10 µm. **(c)** The migration trajectory of ZIF-8 coated nanowire microswimmer in 190 mM PBS solution with 2% H_2O_2. The scale bar is 10 µm.

Supplementary References:

1. Wang, J. *et al.* A Silicon Nanowire as a Spectrally Tunable Light-Driven Nanomotor. *Adv. Mater.*, **29**, 1701451 (2017).
2. Zhu, B. Surface initiated polymerisation for applications in materials science. © Bocheng Zhu, 2012.
3. Hochbaum, A. I., Gargas, D., Hwang, Y. J. & Yang, P. Single Crystalline Mesoporous Silicon Nanowires. *Nano Lett.*, **9**, 3550-3554 (2009).
4. Paxton, W. F., Sen, A. & Mallouk, T. E. Motility of Catalytic Nanoparticles through Self-Generated Forces. *Chem. Eur. J.*, **11**, 6462-6470 (2005).
5. Happel, J. & Brenner, H. *Low Reynolds number hydrodynamics: with special applications to particulate media*, (Springer Science & Business Media, 2012).
6. Lyklema, J. *Fundamentals of Interface and Colloid Science : Solid-liguid interfaces*, (Academic Press: San Diego, 1995).
7. Starov VM, Solomentsev YE. Influence of Gel Layers on Electrokinetic Phenomena: 2. Effect of Ions Interaction with the Gel Layer. *J. Colloid Interface Sci.,* **158**, 166-170 (1993).
8. Yariv, E. & Schnitzer, O. The electrophoretic mobility of rod-like particles. *J. Fluid Mech.*, **719**, R3 (2013).
9. Tanaka, S., Nogami, D., Tsuda, N. & Miyake, Y. Synthesis of highly-monodisperse spherical titania particles with diameters in the submicron range. *J. Colloid Interface Sci.*, **334**, 188-194 (2009).
10. "Counting cells using a hemocytometer", Abcam, www.abcam.com/protocols/counting-cells-using-a-haemocytometer .
